# Investigation of GTP-dependent dimerization of G12X K-Ras variants using ultraviolet photodissociation mass spectrometry†
†Electronic supplementary information (ESI) available. See DOI: 10.1039/c9sc01032g


**DOI:** 10.1039/c9sc01032g

**Published:** 2019-07-15

**Authors:** M. Rachel Mehaffey, Christopher L. Schardon, Elisa T. Novelli, Michael B. Cammarata, Lauren J. Webb, Walter Fast, Jennifer S. Brodbelt

**Affiliations:** a Department of Chemistry , University of Texas at Austin , Austin , TX 78712-0165 , USA . Email: jbrodbelt@cm.utexas.edu ; Tel: +1-512-471-0028; b Division of Chemical Biology and Medicinal Chemistry , College of Pharmacy , University of Texas at Austin , Austin , TX 78712 , USA

## Abstract

Mutations in the GTPase enzyme K-Ras, specifically at codon G12, remain the most common genetic alterations in human cancers. The mechanisms governing activation of downstream signaling pathways and how they relate back to the identity of the mutation have yet to be completely defined. Here we use native mass spectrometry (MS) combined with ultraviolet photodissociation (UVPD) to investigate the impact of three G12X mutations (G12C, G12V, G12S) on the homodimerization of K-Ras as well as heterodimerization with a downstream effector protein, Raf. Electrospray ionization (ESI) was used to transfer complexes of WT or G12X K-Ras bound to guanosine 5′-diphosphate (GDP) or GppNHp (non-hydrolyzable analogue of GTP) into the gas phase. Relative abundances of homo- or hetero-dimer complexes were estimated from ESI-MS spectra. K-Ras + Raf heterocomplexes were activated with UVPD to probe structural changes responsible for observed differences in the amount of heterocomplex formed for each variant. Holo (ligand-bound) fragment ions resulting from photodissociation suggest the G12X mutants bind Raf along the expected effector binding region (β-interface) but may interact with Raf *via* an alternative α-interface as well. Variations in backbone cleavage efficiencies during UV photoactivation of each variant were used to relate mutation identity to structural changes that might impact downstream signaling. Specifically, oncogenic upregulation for hydrogen-bonding amino acid substitutions (G12C, G12S) is achieved by stabilizing β-interface interactions with Raf, while a bulkier, hydrophobic G12V substitution leads to destabilization of this interface and instead increases the proximity of residues along the α-helical bundles. This study deciphers new pieces of the complex puzzle of how different K-Ras mutations exert influence in downstream signaling.

## Abstract 2

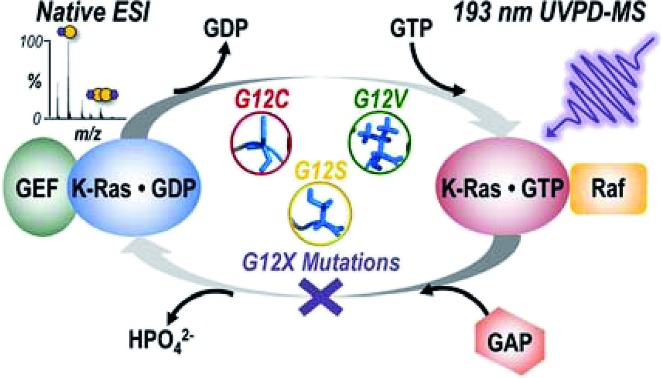
Variations in backbone cleavage efficiencies during UVPD-MS of G12X variants of K-Ras are used to relate mutation identity to structural changes that impact downstream signaling with Raf.

## Introduction

The canonical rat sarcoma (Ras) family of GTPases H-, N-, and K-Ras function as molecular switches and key regulators of cell proliferation and survival through effector pathways, including Raf-MAPK, which communicates signals from surface receptors to the cell nucleus.^1^,^2^ The G-domain (GTPase and effector binding regions) of these proteins is highly conserved while their C-terminal hypervariable regions are post-translationally modified in an isoform specific manner.^1^,^3^–^5^ Ras remains inactive in a GDP-bound state until upstream stimuli cause a switch to the GTP-bound active state, catalyzed by interaction with a guanine-nucleotide exchange factor (GEF). Once activated, Ras binds effector proteins to transmit receptor-initiated signals until it is returned to the inactive state by interaction with a GTPase activating protein (GAP).^1^ Single point mutations most commonly in the Gly12 (G12X) position (or less frequently at the residues specified by codons 13 or 61) prevent GTP hydrolysis, causing the protein to stall in the active state and resulting in activation of cell cycle progression and proliferation.^6^ Consequently, Ras is the most frequently mutated gene in human cancer and an important oncogene for targeting in cancer therapy.^7^,^8^ Based on the knowledge that Ras requires membrane localization and GTP-loading to be active, previous therapeutic strategies involved prevention of membrane localization by inhibiting isoprenylation,^9^ targeting the GTP-binding pocket,^10^–^17^ or interfering with interaction of upstream and downstream effectors.^18^–^21^ However, these efforts to pharmacologically inhibit mutant Ras have met limited success.^22^ Recent evidence from immuno-EM,^23^ spectroscopy,^24^,^25^ dynamic light scattering,^26^ and microscopy^27^–^29^ experiments suggest that Ras functions as a dimer (or less likely as a nanocluster^30^,^31^), not as a monomer. Accordingly, therapeutic efforts have expanded to disruption of the mechanisms modulating Ras oligomerization instead of solely the enzymatic ones.^32^–^36^ The regulation of Ras dimer formation and signaling is still poorly understood, although based on modeling there are two known dimer interfaces along the protein: (1) β-sheet interface at the switch I/effector binding region and (2) α-helix interface at the C-terminal allosteric lobe of the G domain.^26^,^36^ Studies that reveal integral components of Ras dimers, decipher mechanisms regulating formation of these complexes, and define how oncogenic mutations impact these processes are needed to guide the development of more effective therapeutic strategies.

In recent years, mass spectrometry (MS) has proven to be a useful experimental technique to probe theoretical models and address structural biology questions in a sensitive, rapid manner.^37^,^38^ The use of covalent chemical probes, such as hydrogen/deuterium exchange (HDX) or cross-linking reagents, in conjunction with bottom-up tandem mass spectrometry (MS/MS) is now a standard approach for evaluating solvent accessibilities and mapping protein interfaces.^39^–^41^ Top-down techniques have more recently gained traction for characterization of protein structure with the advent of native MS and advances in MS/MS methods.^37^,^38^ The framework for native MS entails the transfer of intact protein complexes to the gas phase by electrospray ionization of solutions of high ionic strength which largely preserves tertiary and quaternary structures of macromolecules.^42^ Consequently, native MS is now routinely used for identification of oligomeric states of proteins and elucidation of binding stoichiometry with other proteins or ligands.^38^ To further discern three-dimensional shapes of protein complexes in the gas phase, ion mobility (IM) mass spectrometry has proved to be an indispensable technique for providing information complementary to static high resolution structures resulting from X-ray crystallography, NMR spectroscopy, or cryo-EM.^43^

MS/MS methods sensitive to protein structure have been developed to study the architecture of protein–protein and protein–ligand complexes. Traditional collisional activation of native-like complexes mainly yields ejection of ligands or subunits,^44^,^45^ which is often uninformative and has spurred the application of alternative activation methods. Electron-based methods, including electron transfer dissociation (ETD) and electron capture dissociation (ECD), and surface induced dissociation (SID) represent the most widely used alternative methods to characterize protein structure.^46^–^52^ ETD and ECD yield mostly diagnostic sequence ions with abundances that can be correlated with crystallographic B-factors and used to probe the topology of protein assemblies for flexible regions, thus revealing insight into the higher order structure of the complexes.^46^–^51^ Conversely, SID causes disassembly of protein–protein complexes into intact subunits in a way that conserves the symmetry of charge distribution among the separating proteins or subunits.^52^ Coupled with IM, this MS/MS technique allows for the rapid determination of stoichiometry and topology for computationally designed protein complexes.^53^ Ultraviolet photodissociation (UVPD) represents a third alternative MS/MS method that utilizes fast, high-energy excitation *via* 193 nm photons and yields the widest array of diagnostic sequence ions for protein complexes.^54^–^58^ The abundances of holo (ligand-bound) and apo (ligand-free) product ions resulting from activation of protein-ligand complexes by 193 nm UVPD have been demonstrated to reflect secondary and tertiary structural features.^59^–^62^ The suppression or enhancement of UVPD at each position along the backbone is thought to be modulated by non-covalent interactions that stabilize structural features and prevent separation of fragments after bond cleavage. Recent studies have demonstrated this approach is sensitive to loop movements upon ligand binding in dihydrofolate reductase (DHFR),^60^ dynamic plasticity throughout a catalytic cycle of the active enzyme adenylate kinase,^61^ and even long-range conformational changes resulting from single residue mutations in K-Ras.^62^

Previously variations in UVPD backbone cleavage efficiencies were monitored for monomeric protein-ligand complexes of K-Ras (wild-type (WT) and three G12X mutants: G12C, G12V, G12S) upon exchange of GDP for a non-hydrolyzable GTP analogue, guanosine 5′-[β,γ-imido]triphosphate (GppNHp).^62^ Switching from the inactive diphosphate bound state to the active triphosphate bound protein yielded unique structural changes for each mutant. Based on the examination of monomeric K-Ras and a series of G12X variants, the way each mutation modulated homo- or hetero-dimer formation with downstream effectors was inferred. Given that the G12 position lies on the surface of the protein, longer-range conformational changes in areas of the protein related to dimer formation are likely involved. Specifically, observations included stabilization of the α-helical bundle for G12C K-Ras, stabilization of the β-sheet region for G12V K-Ras, and increased flexibility of the β-sheet region for G12S K-Ras.^62^ Although the mechanism is not yet well defined, this supports that the identity of the G12 substitution impacts downstream signaling and patient survival differently.^63^–^69^

Here we report the use of native MS and UVPD to directly interrogate homo- and hetero-dimers of WT and three clinically relevant G12X mutants of K-Ras (G12C, G12S, G12V). We use native MS to define the relative abundance of homodimer formation for each K-Ras G12X mutant bound to either GDP or GppNHp (the non-hydrolyzable analogue of GTP), and compare the amount of heterodimer formed between each of the K-Ras mutants and the Ras binding domain (RBD) of a downstream effector kinase, rapidly accelerated fibrosarcoma (Raf). Comparing UVPD cleavage efficiencies of K-Ras·GppNHp + Raf_RBD_ heterodimers for each of the mutants to the WT suggests hydrogen-bonding amino acid substitutions (G12C, G12S) rely on directly stabilizing the β-interface interactions with Raf for oncogenic upregulation, whereas a bulkier, hydrophobic G12V substitution causes destabilization of this interface and instead results in tightened α-helical bundles. Direct structural interrogation of intact dimers by UVPD-MS represents an advance in unraveling the mutation-dependent interplay of structure and binding interactions for K-Ras signaling phenotypes.

## Experimental

### Sample preparation

Four variants of recombinant human K-Ras (WT, G12C, G12V, G12S) were expressed and purified as previously described.^62^ The RBD of c-Raf-1 was expressed using a purchased plasmid and purified as described in the ESI.† Expressed sequences of K-Ras and Raf, and structures of the guanosine phosphate ligands GDP and GppNHp (the stable GTP analogue) are shown in Fig. S1.† Given that purified K-Ras was already bound to GDP, the procedure for nucleotide exchange to GppNHp is included in the ESI.† Protein samples were diluted to 20 μM or 80 μM dimer concentration in 50 mM ammonium acetate containing 5 μM magnesium acetate (pH 7.8) and desalted for MS analysis using 3 kDa molecular weight cutoff filter devices (EMD Millipore, Billerica, MA). For heterodimer experiments, K-Ras (∼19.3 kDa) and Raf (∼9.4 kDa) were combined at a 1 : 1 ratio before desalting.

### Mass spectrometry

Experiments were performed on a Thermo Scientific Orbitrap Elite mass spectrometer (Bremen, Germany) modified with a 193 nm Coherent Excistar excimer laser (Santa Cruz, CA) to allow photodissociation in the HCD cell.^54^ An offline nano-ESI source with Au-coated borosilicate emitters ionized the native complexes using source voltages of 1.0–1.1 kV and a source temperature of 200 °C. A resolving power of 240 K at *m*/*z* 400 was used to collect all MS spectra. MS1 spectra represent sixty scans with an automated gain control (AGC) setting of 1 × 10^6^. Details for on-line size-exclusion chromatography experiments of K-Ras·GppNHp + Raf_RBD_ complexes are given in the ESI.† MS/MS analysis of K-Ras·GppNHp + Raf_RBD_ heterodimers involved selection of the 12+ charge state of the complex using an isolation width of 20 *m*/*z* and activation with a single 3 mJ pulse. Each UVPD mass spectrum represents 500 transients with a scan range *m*/*z* 220–4000 using an AGC value of 5 × 10^5^ and maximum ion time of 2 s. For each K-Ras·GppNHp + Raf_RBD_ heterocomplex triplicate UVPD data was collected.

### Data analysis

For MS1 spectra, the amount of K-Ras homodimer formation was assessed by normalizing the summed abundances of all charge states corresponding to the observed dimer to the total ion current of the spectrum. UVPD MS/MS spectra were de-charged and de-isotoped using the Thermo Xtract algorithm (S/N ratio of 3, fit factor of 44%, remainder of 25%). Monoisotopic apo fragment ions were identified using ProSight Lite v1.4 which accounts for the nine ion types typically observed during UVPD (a, a + 1, b, c, x, x + 1, y, y – 1, z). Holo fragment ions were also identified by including mass shifts in the search corresponding to the GppNHp ligand, coordinating Mg^2+^ ion, and intact Raf_RBD_ subunit. Although Raf_RBD_ itself likely underwent fragmentation during UV activation, to streamline data interpretation this subunit was treated as a ligand bound to K-Ras and only the intact mass shift of Raf_RBD_ was searched. Confident identification of holo fragment ions containing a cleaved portion of K-Ras bound to a cleaved portion of Raf_RBD_ is impeded given the nearly unlimited array of possible fragment ion assignments that can arise from combinations of partial protein sequence segments. Thus, assignment of these types of holo ions was not considered owing to low statistical confidence. Given that Raf_RBD_ is much smaller than K-Ras and has a different sequence, the unidentified fragments of Raf_RBD_ should not overlap significantly with the K-Ras fragments of interest. Specific mass additions included in the searches for holo ions are: 9952.879–9954.894 Da for Raf_RBD_ + GppNHp·Mg^2+^, and 542.969–544.984 Da for GppNHp·Mg^2+^ alone. The divalent Mg^2+^ cofactor necessary to coordinate the GppNHp ligand contributed a +21.969 Da shift. The fragment abundance utility of UV-POSIT^70^ was used to sum cleavage yields at each backbone position upon UVPD if the amino acids of a protein are numbered from 1 (N-terminus) to R (C-terminus) for a protein containing R amino acids. This web-based program normalizes the abundances of identified holo and corresponding apo ions to the total ion current of the spectrum by collectively summing N-terminal product ions (a_*n*_, b_*n*_, c_*n*_) arising from backbone cleavage C-terminal to a given amino acid, *n*, with C-terminal fragment ions (x_R-*n*+1_, y_R-*n*+1_, z_R-*n*+1_) resulting from cleavage N-terminal to residue *n*.^70^ This calculated value is used to convey the UVPD backbone cleavage efficiency adjacent to each amino acid throughout the protein. Student's *t*-test with pooled standard deviations was used to determine the statistical significance of differences in backbone cleavage efficiency upon UVPD for WT and G12X K-Ras·GppNHp + Raf_RBD_ heterocomplexes. A two-tailed test was assumed to determine *p*-values from calculated *t*-values. Fig. S2† gives graphs of the log of calculated *p*-values per residue for backbone cleavage efficiency of heterodimers of each G12X K-Ras variant compared to WT. The black dotted line represents a confidence threshold at 99%. A histogram of calculated *p*-values for the entire data set is shown in Fig. S2† demonstrating over 55% of measured UVPD backbone cleavage efficiencies within a triplicate measurement are statistically different for G12X variants from the measured average of the corresponding WT backbone position at the 99% confidence level. For reference, Fig. S3† is the crystal structure of WT K-Ras bound to GppNHp and complexed with Raf_RBD_ (PDB ID: ; 4G0N)^71^ with important helical and loop regions labelled and the two dimer interfaces highlighted.

Owing to the fact that UVPD-MS is a new approach for characterization of structural variations in protein complexes and providing insight into potential changes in conformation and binding motifs, there is need for critical evaluation of the method *via* appropriate controls and statistical tests. Some of the statistical tests and controls are described above and shown in Fig. S2, S9 and S16.† Moreover, the K-Ras protein used in the present study was also the subject of a prior study (ref. 62). Additional controls and statistical tests were performed previously, as provided in Fig. S2–S4† in ref. 62, including the assessment of the statistical significance of backbone cleavage efficiency for K-Ras and the G12C, G12V, and G12S mutants, both as native-like and denatured forms. One relevant control entailed the examination of UVPD of the denatured proteins, confirming that the relative fragment ion abundances were identical for 166 out of 169 comparisons of specific backbone positions. In contrast, the UVPD fragment ion abundances of the native-like proteins showed numerous statistically significant differences in fragment ion abundances. These types of control experiments support the premise that UVPD is sensitive to protein structure.

## Results & discussion

### Native MS to detect GTP-dependent formation of K-Ras homodimers

In addition to membrane localization and GTP-loading, recent studies posit the formation of homodimers as a significant factor in signaling output related to K-Ras.^23^–^29^ Specifically, dimerization of K-Ras might promote self-association of downstream effectors necessary for their activation.^23^,^28^ Consequently, defining the extent to which specific G12X mutations impact the formation of K-Ras homodimers when bound to either GDP or GTP is essential. ESI-mass spectra were collected under native conditions for WT, G12C, G12V, and G12S K-Ras sprayed with a 1 : 1 ratio of either GDP or GppNHp (the non-hydrolyzable analogue of the GTP ligand) at both a relatively standard (20 μM) and high (80 μM) protein concentration (Fig. S4†). In addition to the ligand-bound monomers (9+, 10+, 11+), homodimers (12+, 13+, 14+) were observed for all K-Ras protein except G12V at 20 μM. Table S1† summarizes the measured intact masses for observed dimers containing two protein molecules and two ligands. Native MS represents a unique strategy to determine both stoichiometries and compositions of complexes. The experimentally measured masses confirm that each subunit of the dimer is bound to the same ligand (GDP or GppNHp) as the other subunit (since the nucleotide exchange is 90–95% efficient, there is always a portion of GDP-bound K-Ras in solution). Other biochemical techniques have been used to demonstrate the existence of K-Ras dimers but lacked sufficient mass resolution to confirm their specific compositions.^23^–^29^

**Fig. 1:**
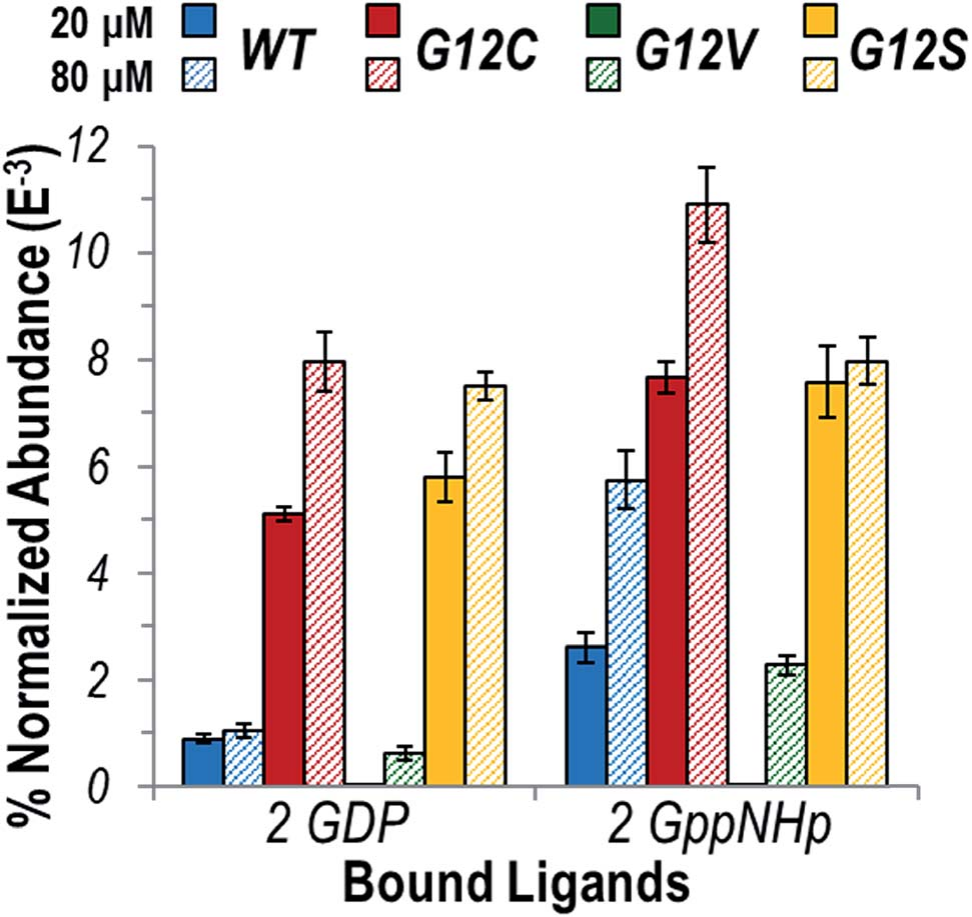
Graph displaying the abundance of each K-Ras homodimer normalized to the total ion current (TIC) for the native ESI mass spectra (Fig. S4†) for WT (blue), G12C (red), G12V (green), and G12S (yellow) bound to two GDP or two GppNHp (1 : 1 ratio) at a protein concentration of 20 μM (solid bars) or 80 μM (hashed bars). GppNHp is a non-hydrolyzable analogue of GTP and represents the active state ligand of K-Ras. The theoretical and measured monoisotopic masses for each observed homodimer are reported in Table S1.†

The distribution of homodimers *versus* monomers for each variant are estimated by comparing normalized abundances of the ions in the native ESI mass spectra shown in Fig. S4.† Fig. 1 illustrates the relative abundances of each observed K-Ras homodimer normalized to the total ion current for WT, G12C, G12V, and G12S K-Ras bound to GDP or GppNHp for protein concentrations of 20 or 80 μM. A larger bar indicates a greater portion of the dimeric form of the protein relative to the monomeric protein. GppNHp-bound WT K-Ras was observed to form significantly more abundant homodimers compared to its GDP-bound counterpart. This result is in line with the significant role of dimerization in the activation of K-Ras. The same general trend indicating enhanced formation of dimers containing GppNHp over GDP was observed for both G12C and G12S K-Ras, and for each of these two variants the overall abundances of homodimers containing GDP or GppNHp were substantially higher than the abundances of WT homodimers. Since the G12C and G12S mutations result in constitutively active variants of K-Ras, higher abundances of the homodimers support the idea that oligomerization is important for activation of the enzyme. Interestingly, the abundances of G12V K-Ras homodimers were low in general and were only observed at the higher (80 μM) protein concentration, suggesting dimerization is not a significant factor in maintaining an active state when the bulky Val is substituted for Gly.

In general, the relatively low percentage of dimers (<1% to 11% in Fig. 1) compared to the corresponding monomers is not surprising given that the protein is in solution with no membrane localization to aid in self-assembly.^23^,^28^ Previous dynamic light scattering experiments observed higher order oligomers only for K-Ras bound to another GTP-analogue, guanosine 5′-O-[γ-thio]triphosphate (GTP-γ-S), not GDP nor GppNHP.^26^ Although GTP-γ-S is expected to be a better mimic of GTP, for the experimental conditions tested herein this compound hydrolyzed too quickly to allow confident measurements of protein complexation (Fig. S5†).

There is accumulating evidence that WT K-Ras can act as a tumor suppressor and counteract the activating effects of G12X mutations.^72^ To mimic cells heterozygous for G12X mutations, each of the three mutants bound to GDP or GppNHp was mixed in a 1 : 1 ratio with WT K-Ras at 20 or 80 μM total protein concentration prior to ESI. No dimers were observed in the ESI-MS spectra, thus suggesting that homodimers composed of two protein variants (WT and G12X K-Ras) are less stable or membrane localization is required for formation (Fig. S6†).

### Impact of G12X mutations on K-Ras:Raf heterodimer formation

**Fig. 2:**
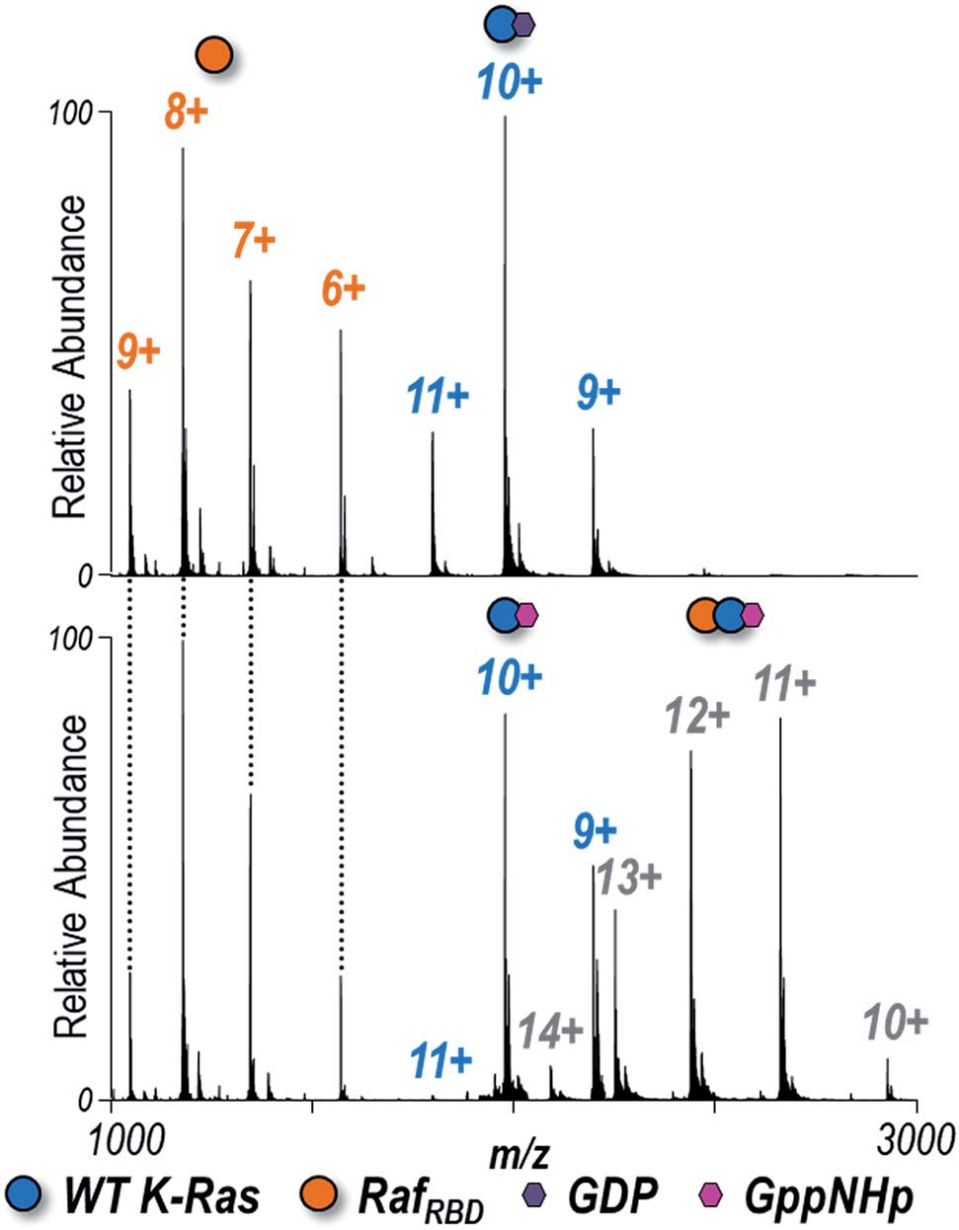
Native ESI mass spectra of 1 : 1 K-Ras : Raf_RBD_ solution of WT K-Ras bound to (A) GDP or (B) GppNHp (1 : 1 ratio). Observed species are labelled as colored circles (6+, 7+, 8+, 9+ Raf_RBD_ monomers, 9+, 10+, 11+ K-Ras monomers, and 10+, 11+, 12+, 13+, 14+ heterodimers). Corresponding spectra for G12C, G12V, and G12S K-Ras are shown in Fig. S7.† K-Ras:Raf_RBD_ heterodimers were only observed for solutions containing GppNHp (non-hydrolyzable analogue of GTP), not GDP. The relative abundances of each species are estimated from SEC-MS and reported in Fig. 3. The 12+ heterodimer was selectively isolated and activated to yield the UVPD spectra shown in Fig. S10.†

Understanding how specific G12X mutations in K-Ras affect interactions with downstream effectors is another piece of the puzzle encompassing the mechanism by which individual substitutions impact signaling.^65^ Each of the four K-Ras variants bound to either GDP or GppNHp (the non-hydrolyzable analogue of GTP) was combined in a 1 : 1 ratio with the Ras binding domain of Raf (Raf_RBD_) to determine the extent to which heterocomplexes are formed in solution. ESI-MS spectra are shown for WT K-Ras in Fig. 2 and for the three G12X variants in Fig. S7.† K-Ras:Raf_RBD_ heterodimers were observed only for GppNHp-bound K-Ras but not for GDP-bound K-Ras (Fig. 2), a result consistent with the fact that only activated K-Ras (GTP-bound form) should bind Raf_RBD_. The same outcome is true for the formation of heterodimers of the G12X variants in Fig. S7:† G12X:Raf_RBD_ heterodimers were only observed for solutions containing GppNHp, not GDP.

**Fig. 3:**
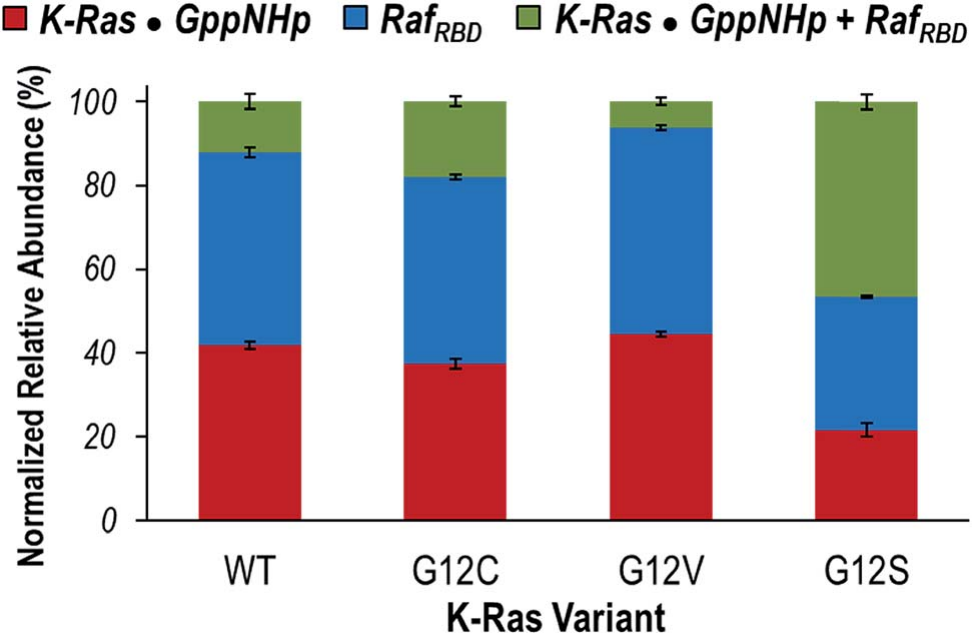
Distribution of the normalized relative abundances of K-Ras·GppNHp (red), Raf_RBD_ (blue), and K-Ras·GppNHp + Raf_RBD_ heterodimer (green) from on-line SEC of solutions containing WT, G12C, G12V, or G12S K-Ras·GppNHp with an equimolar amount of Raf_RBD_. Peak areas of the EIC traces of *m*/*z* values corresponding to each species were used to calculate the reported percentages (Fig. S8†).

Given that the observed monomeric and dimeric protein complexes span a broad *m*/*z* range, on-line size-exclusion chromatography was used to separate the various complexes in order to allow optimization of MS tuning parameters and improve the accurate measurement of abundances. Fig. S8† illustrates extracted ion chromatograms (EICs) derived for the *m*/*z* values corresponding to each key species (K-Ras·GppNHp, Raf_RBD_, and K-Ras·GppNHp + Raf_RBD_). Peak areas from the EICs were used to estimate the distributions of the monomeric proteins (K-Ras·GppNHp and Raf_RBD_) and heterodimers (K-Ras·GppNHp + Raf_RBD_) for WT K-Ras and each of the three variants (Fig. 3). The portions of K-Ras·GppNHp + Raf_RBD_ heterodimers (green segments) are the focal point of Fig. 3. G12V yields a significantly lower percentage of heterodimers than WT K-Ras, and conversely G12C and G12S display a larger percentage of heterodimers than WT K-Ras. This general trend in the formation of K-Ras·GppNHp + Raf_RBD_ heterodimers mirrors the results reported above for K-Ras homodimer formation. These results suggest that amino acid substitutions (G12C, G12S) that have enhanced hydrogen-bonding capabilities contribute to stabilizing the β-interface interactions with Raf. In contrast, the bulkier, hydrophobic G12V substitution causes destabilization of this interface.

### Analysis of UVPD holo fragment ions to examine K-Ras:Raf heterodimer interface

To further probe the differences in protein–protein interactions governing the binding of WT and G12X K-Ras to Raf_RBD_, the most abundant charge state (12+) of each observed heterocomplex (K-Ras·GppNHp + Raf_RBD_) was subjected to 193 nm UV photoactivation. Deconvoluted mass spectra of the isolated precursor ions are shown in Fig. S9,† confirming that the populations of heterocomplexes are similar for WT K-Ras and all three variants. Fig. S10† displays the UVPD MS/MS spectra for all four K-Ras·GppNHp + Raf_RBD_ complexes. In addition to disassembly of the complexes into their constituent subunits (K-Ras·GppNHp (9+) and Raf_RBD_ (3+)), UVPD also yields diagnostic sequence ions.^56^ Deconvolution of the UVPD spectra allows identification of these fragment ions (Fig. S11†). Based on the fragmentation patterns from UVPD, sequence coverage maps for each K-Ras variant are given in Fig. S12.† UVPD afforded 85–89% coverage of WT K-Ras or G12X for each of the four complexes.

**Fig. 4:**
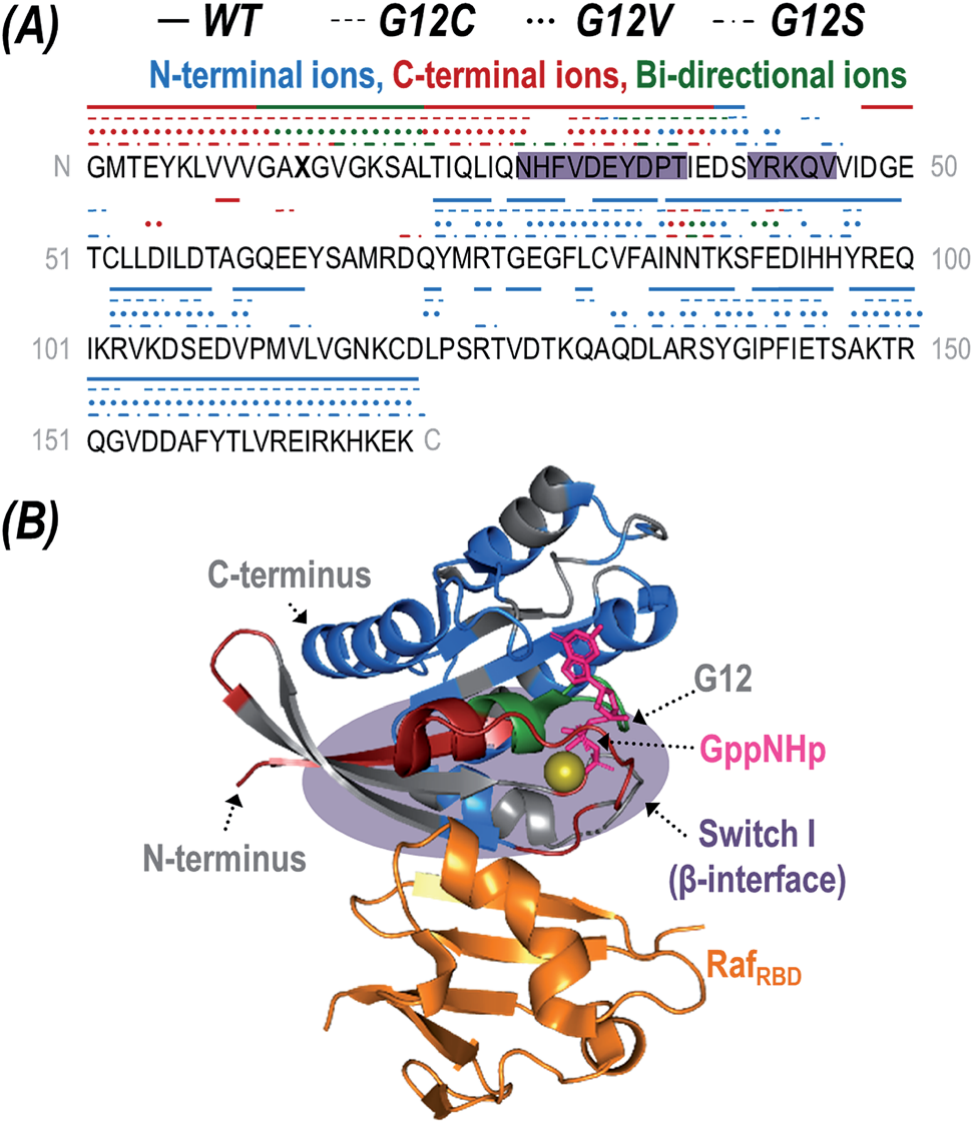
(A) The backbone cleavage sites upon UVPD of the K-Ras·GppNHp + Raf_RBD_ heterodimer (12+) are mapped above the sequence of K-Ras (X13 = G, C, V, or S) as lines or symbols for WT (solid), G12C (dashes), G12V (dots), and G12S (pattern). The backbone cleavages that result in N-terminal ions (*a*,*b*,*c*) are colored blue; those that result in C-terminal ions (*x*,*y*,*z*) are colored red; and those that yield complementary N-terminal and C-terminal ions are categorized as bi-directional and are colored green. The β-interface region is shaded in purple. (B) Crystal structure of K-Ras bound to GppNHp and complexed with Raf_RBD_ (PDB ID: ; 4G0N) with residues colored corresponding to N-terminal (*a*,*b*,*c*-type ions; blue), C-terminal (*x*,*y*,*z*-type ions; red), and bi-directional (green) holo fragment ions observed during UVPD of the 12+ WT heterodimer. Corresponding structures for the G12X mutants are given in Fig. S13.† The original UVPD spectra are shown in Fig. S10.† The GppNHp ligand (a non-hydrolyzable analogue of GTP) is shown as pink sticks and labelled in (B).

The fragment ions generated upon UVPD can be further categorized as apo (Raf_RBD_-free) or holo (containing Raf_RBD_) product ions. While traditional HCD typically causes ejection of the intact ligand prior to backbone fragmentation owing to the preferential cleavage of non-covalent interactions during collisional activation, UV photoactivation of protein-ligand complexes yields diagnostic sequence ions that retain non-covalent interactions with the bound ligands, termed holo fragment ions.^55^,^60^–^62^,^73^ HCD has been reported to yield holo ions consisting of a portion of a non-covalently bound nucleotide^74^ but UVPD consistently allows retention of intact nucleotides like GTP or GppNHp.^62^ The high energy imparted to a protein *via* absorption of 193 nm photons results in activation to excited electronic states and affords preferential cleavage of backbone bonds rather than disruption of electrostatic interactions with bound ligands. Consequently, mapping the location of observed holo ions along the protein backbone affords insight into ligand-binding interfaces. Fig. 4A displays the sequence of K-Ras with indicators above specific residues to show the sites of backbone cleavages that lead to production of holo ions. The indicators are color-coded to reflect whether the backbone cleavage sites correspond to formation of N-terminal ions (*a*,*b*,*c*), C-terminal ions (*x*,*y*,*z*), or bi-directional ions (*i.e.* backbone cleavages resulting in complementary N- and C-terminal holo ions). Although UVPD of the K-Ras·Raf_RBD_ complexes caused backbone fragmentation of both the K-Ras and Raf_RBD_ subunits, for streamlined data interpretation Raf_RBD_ was treated as one large ligand and only holo ions containing the intact Raf_RBD_ protein were considered. The crystal structure of K-Ras bound to Raf_RBD_ is shown in Fig. 4B with residues corresponding to the observed sites of backbone cleavages color coded to aid in visualization for WT K-Ras (and corresponding structures for the three G12X mutants given in Fig. S13†).

Of the two known interfaces along K-Ras (α- and β-interfaces), effector binding is expected to occur at the stronger β-interface containing Switch-I (residues 27–36, 41–45, shaded in Fig. 4A).^26^ The observed backbone cleavage sites that lead to Raf_RBD_-bound holo ions upon UVPD of the K-Ras·Raf_RBD_ complexes support the β-interface as the primary binding region for WT K-Ras as well as for the three G12X mutants. In particular, backbone cleavages that result in C-terminal (*x*,*y*,*z*) holo ions only occur in the region of K-Ras that is N-terminal to the β-interface, and backbone cleavages that lead to N-terminal (*a*,*b*,*c*) ions occur exclusively in the region C-terminal to the β-interface (Fig. 4A). Moreover, the bi-directional holo ions that result in complementary C-terminal and N-terminal product ions originate from backbone cleavages along or near this expected interface. Further evidence is provided by the apo sequence coverage maps (Fig. S12†) in which coverage of the β-interfacial region (shaded in purple) is relatively sparse, suggesting two possibilities. One possibility is that backbone cleavages in the β-interfacial region might instead preferentially produce the mass-shifted holo fragment ions showcased in Fig. 4A (thus depleting the abundances of apo sequence ions). Alternatively, fragmentation along the β-interfacial region might be suppressed owing to its engagement in interactions with Raf_RBD_ (thus stabilizing the β-interfacial region and preventing separation of fragment ions).

Interestingly, there are several C-terminal and bi-directional holo fragment ions originating from backbone cleavages adjacent to residues N86-E92 (C-terminal to the β-interface) which are observed for all three G12X mutants but not WT K-Ras (Fig. 4A). This region is part of the weaker α-interface (residues 86–105, 126–138) where K-Ras homodimerization is thought to occur.^16^,^26^ Perhaps G12X mutants also use the α-interface to bind effector proteins and maintain an active state. However, the fact that fewer holo ions originate from this region suggests either it is a lower population effector binding region compared to the β-interface or the non-covalent interactions stabilizing this alternative binding mode are too weak to survive photodissociation (thus preventing survival of holo ions). The observations about the involvement of the α-interface merit future investigation using other traditional biophysical methods.

### Variations in UVPD cleavage efficiency for G12X K-Ras:Raf heterodimers

**Fig. 5:**
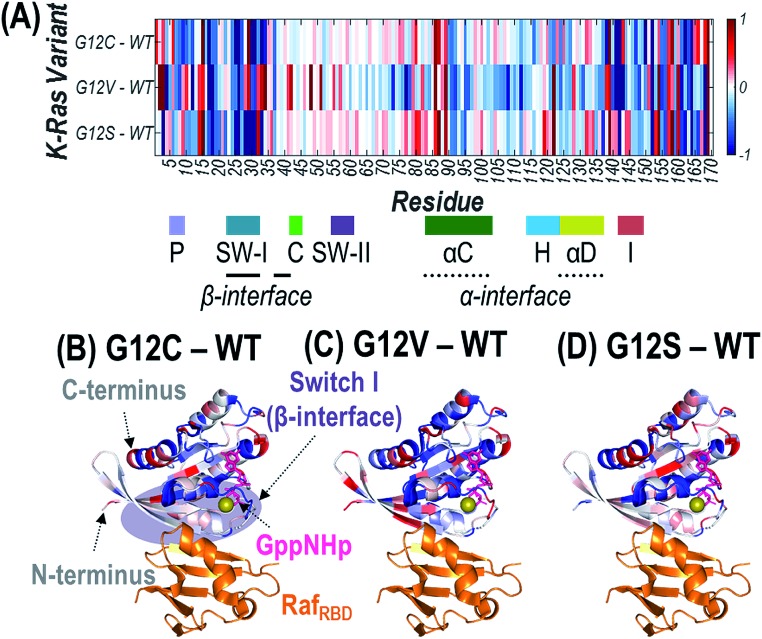
Heat maps of the enhancement (red) and suppression (blue) of UVPD for Raf_RBD_-bound heterodimers shown as a linear map across the sequence (A) or displayed along the crystal structure of K-Ras bound to GppNHp (non-hydrolyzable analogue of GTP) and complexed with Raf_RBD_ for (B) G12C, (C) G12V, and (D) G12S K-Ras variants relative to WT K-Ras. These heat maps are difference plots: (*i.e.* G12X – WT). The colored regions represent statistically significant changes in cleavage efficiency of the protein backbone during UVPD based on the difference plots in Fig. S14.† The GppNHp ligand (pink) is shown as sticks and labelled in (B). The β-interface region is highlighted in purple in (B). Relevant loops, α-helices, and interfaces are labelled underneath the *x*-axis of (A).

Since the mutated G12 position is located along the outer surface of K-Ras, it is expected that the conformational changes induced by the substitution result in longer-range changes in regions of the protein involved in dimer formation.^62^ UVPD has previously been demonstrated to be sensitive to these types of structural changes for other protein complexes.^59^–^62^ Specifically, variations in the efficiency of backbone cleavage induced by UVPD relative to the same protein in a different state are determined for each residue. The extent to which a given region engages in stabilizing intramolecular interactions (*i.e.* protein organization and rigidity) can be inferred from enhancement or suppression in UVPD backbone cleavage efficiency.^59^–^62^ Details are provided in the Experimental section on how backbone fragment ion abundances are summed and how cleavage efficiency is determined to generate the UVPD backbone cleavage efficiency plots shown in Fig. S14.† Comparisons of the backbone cleavage efficiencies of G12X mutants to WT K-Ras are best visualized as plots of the differences in UVPD backbone cleavage efficiency for each residue (*i.e.* backbone cleavage efficiency of WT subtracted from the backbone cleavage efficiency of each mutant at each position) (Fig. S15†). A histogram of all *p*-values calculated for *t*-test comparisons corresponding to the difference plots in Fig. S15† is shown in Fig. S2.† The differences in backbone cleavage efficiencies are represented as a heat map in which suppression of UVPD (values that fall below the zero axis) or enhancement of UVPD (values that lie above the zero axis) compared to UVPD of WT K-Ras are highlighted in blue and red, respectively (Fig. 5A). The heat map values are imprinted on the crystal structure of K-Ras·GppNHp + Raf_RBD_ to aid in relating conformational changes to structural features of the protein (Fig. 5B–D).

To gain insight into how the identity of the G12X mutations impacts the effects on signaling, we focus on conformational differences observed in three key regions: β-interface (switch I: residues 27–36, 41–45), switch II (residues 58–64), and α-interface (α-C and α-D helices: residues 86–105 and 126–138). The three key regions of the difference plots shown in Fig. S15† are expanded in Fig. S16,† and the statistically meaningful variations at the 99% confidence level (*p* ≤ 0.01) are demarcated to help highlight the trends in UVPD backbone cleavage efficiencies for each of the three variants compared to WT K-Ras. Fig. S17† summarizes the observed enhancement or suppression in UVPD backbone cleavage efficiency of those three regions for Raf_RBD_-bound heterodimers of G12C, G12V, and G12S compared to WT K-Ras. G12C and G12S result in similar changes in these regions, mainly stabilization of the β-interface (*e.g.*, suppression of UVPD). Conversely the G12V substitution favors increased proximity of the helices in the α-interfacial region (observed as suppression of UVPD) and destabilization of the β-interface (resulting in enhancement of UVPD).

These observations bring new light to a previous study that used UVPD-MS to evaluate conformational changes that occurred for K-Ras and its three G12X mutants during the GTP loading step of K-Ras activation.^62^ In the previous study, a more stable α-helical region was inferred for G12C K-Ras in comparison to WT K-Ras based on suppression of UVPD in that region, whereas G12S K-Ras exhibited more flexibility in the β-interface region based on enhancement of UVPD.^62^ Both of these findings are consistent with the adoption of a more stable β-interface upon Raf_RBD_ binding in the present study (*i.e.* given the β-interface was found to be more flexible, more intramolecular contacts could be formed upon Raf binding). For G12V K-Ras compared to WT K-Ras, the converse was observed previously: the β-interface appeared to be more rigid (stabilized by more interactions; lower UVPD fragmentation efficiency) after substitution of GppNHp for GDP.^62^ Again this finding correlates with the more stabilized α-interface of G12V after Raf_RBD_ binding as evidenced by the decreased UVPD fragmentation efficiency in the present study. This outcome implies engagement of fewer interactions between Raf_RBD_ and the β-interface, since G12V K-Ras was more rigidly pre-organized prior to effector binding. Collectively these results suggest that mutations of K-Ras which introduce hydrogen-bonding groups (G12C, G12S) result in a stabilized β-interface, although it is also acknowledged that thiols form weaker hydrogen bonds than alcohols.^75^ In contrast, a bulkier, more hydrophobic substitution (G12V) relies preferentially on contacts along the α-interface for oncogenic activation of K-Ras.

## Conclusion

Native MS and UVPD-MS were used to probe homo- and hetero-dimers of WT and G12X mutants of K-Ras, along with its effector protein Raf. K-Ras formed more homodimers when bound to a GTP-analogue compared to GDP for the WT, G12C, and G12S variants. G12V K-Ras only formed homodimers at a relatively high protein concentration. Similar results were observed for heterodimer formation between K-Ras and the Ras binding domain of effector protein Raf: compared to WT K-Ras, G12V K-Ras yielded less abundant heterodimers in contrast to G12C and G12S K-Ras. Characterization of the K-Ras·GppNHp + Raf_RBD_ heterocomplexes by UVPD revealed that all three G12X mutants prefer binding along the β-interface, which is the expected effector binding region for WT K-Ras. However, there is also evidence that the three mutants, but not WT K-Ras, can bind Raf along the weaker α-interface as well. Moreover, comparison of UVPD backbone cleavage efficiencies for the G12X mutants relative to WT K-Ras afforded insight into longer-range conformational changes responsible for observed differences in downstream signaling. Specifically, the G12C and G12S substitutions (ones that introduce hydrogen-bonding groups) resulted in a stabilized β-interface, whereas the G12V mutation (a bulky, hydrophobic substitution) yielded tighter helical bundles along the α-interface. G-domain non-covalent interactions (α- and β-interfaces) are only one of the factors governing homo- and hetero-dimerization, along with membrane localization by post-translational modifications along the hypervariable region of K-Ras or other scaffold proteins. This work offers new insight into the seemingly complex mechanism relating the identity of G12X mutations to different downstream effects.

## Conflicts of interest

There are no conflicts to declare.

## Supplementary Material

Supplementary information
