# Supplementary material for: Investigation of GTP-dependent dimerization of G12X K-Ras variants using ultraviolet photodissociation mass spectrometry
Source: Chem Sci. 2019 Jul 15;10(34):8025–34. doi: 10.1039/c9sc01032g (PMC6837035; doi:10.1039/c9sc01032g)

Electronic Supplementary Material (ESI) for Chemical Science.
This journal is © The Royal Society of Chemistry 2019

Investigation of GTP-Dependent Dimerization of G12X K-Ras Variants Using Ultraviolet Photodissociation Mass Spectrometry

M. Rachel Mehaffey[1], Christopher L. Schardon[2], Elisa T. Novelli[1], Michael B. Cammarata[1], Lauren J. Webb[1], Walter Fast[2], Jennifer S. Brodbelt[1*]

[1]Department of Chemistry and [2]Division of Chemical Biology and Medicinal Chemistry, College of Pharmacy, University of Texas at Austin, Austin, TX 78712

Corresponding author: Jennifer S. Brodbelt
University of Texas at Austin
Department of Chemistry
Austin, TX 78712-0165
jbrodbelt@cm.utexas.edu
Phone: (512)-471-0028

Supporting Information:
Supporting information contains detailed descriptions of experimental methods used to express and purify proteins, exchange nucleotides, and collect on-line size-exclusion chromatography. Figures S1-S17 include the expressed protein sequences of K-Ras and Raf, structures of the guanosine phosphate ligands, plots of p-values demonstrating statistical significance, crystal structure of K-Ras bound to Raf with important regions labelled, MS1 of K-Ras•GppNHp homodimers and K-Ras•GppNHp + Raf heterodimers, ESI-MS of GppNHp ligand, extracted ion chromatograms from size-exclusion chromatography of K-Ras•GppNHp + Raf heterodimers, UVPD MS/MS spectra of K-Ras•GppNHp + Raf heterodimers, apo fragment ion sequence coverage maps for UVPD spectra of K-Ras•GppNHp + Raf heterodimers, crystal structures mapping observed holo fragment ions, UVPD fragment ion intensity and difference plots for K-Ras•GppNHp + Raf heterodimers, and a summary of observed differences in UVPD backbone cleavage efficiency in three key regions for each K-Ras variant compared to WT. Table S1 gives the theoretical and experimental intact masses for K-Ras•GppNHp homodimers.

EXPERIMENTAL

Protein Expression and Purification

A pET 15-b vector containing the gene for the Ras Binding Domain (RBD) of c-Raf-1 with an N-terminal 6X-Histidine tag separated by a thrombin cleavage site was purchased from Genscript. The vector was transformed into BL21(DE3) competent *E. coli* cells (New England Biolabs) for expression. Single colonies of *E. coli* containing the desired plasmid were inoculated in 5 mL of Luria-Bertani (LB) Broth and grown at 37 °C for 14-16 hours. The 5 mL cultures were used to seed 2 L flasks containing Terrific Broth (TB, Millipore) which were incubated with shaking at 37 °C for 4-6 hours until an OD_{600} ~0.6 was obtained. Isopropyl β-D-1-thiogalactopyranoside (IPTG, GoldBio) was added to a final concentration of 1 mM, and protein expression was carried out at 30 °C while shaking for 6 hours.

Cells were collected via centrifugation at 6000 RPM for 15 minutes, and re-suspended in lysis buffer containing 50 mM phosphate, 500 mM NaCl, 40 mM imidazole, and 10% glycerol at pH = 8.0. Re-suspended cells were lysed by sonication with a probe sonicator for 3 rounds consisting of 1 minute of pulsed sonication followed by 5 minutes of resting on ice, for a total sonication time of 3 minutes. The cell debris was separated via centrifugation at 19000 RPM for 30 minutes, and the supernatant was passed through a 10 μM filter then added to an Ni-nitrilotriacetic acid (Ni-NTA) column pre-equilibrated with lysis buffer for affinity chromatography. The flow through was reapplied to the column and then the column was washed with lysis buffer prior to elution with a buffer containing 50 mM phosphate, 500 mM NaCl, 500 mM imidazole, and 10% glycerol at pH = 8.0. The eluent containing the target protein was exchanged into a cleavage buffer containing 20 mM tris, 150 mM NaCl, 2.5 mM $CaCl_2$, and 10% glycerol at pH = 8.0. Protein concentration was estimated via absorbance at 280 nm and thrombin (Millipore) was added to a concentration of 2 U thrombin per mg Raf and incubated overnight at 4 °C. The cleaved Raf was separated from thrombin by applying the protein solution to a benzamidine column equilibrated in cleavage buffer, and the flow through containing Raf was collected. The protein was then exchanged into a final buffer of 50 mM ammonium acetate (pH 7.8). Typical yield was 20-30 mg per liter of growth.

K-Ras Nucleotide Exchange

To remove GDP and load K-Ras with the non-hydrolyzable GTP mimic GppNHP, variants were diluted to 30 μM in an exchange buffer (pH 8.0) consisting of 25 mM Tris, 10 mM EDTA, 1

mM dithiothreitol (DTT), and 1.5 mM guanosine 5'-[β,γ-imido]triphosphate (GppNHp, Sigma Aldrich, St. Louis, MO). This solution was incubated on ice for 1 hour. Subsequently magnesium acetate was added at a final concentration of 25 mM and the mixture was incubated on ice for an additional 30 min. These steps were repeated once after using a 10 kDa molecular weight cutoff filter device (EMD Millipore, Billerica, MA) to put the protein back into exchange buffer. Afterwards, a final buffer exchange was performed to put the GppNHp-loaded protein into MS spray buffer (50 mM ammonium acetate with 5 μM magnesium acetate, pH 7.8).

Size-Exclusion Chromatography

Size-exclusion chromatography experiments were performed by interfacing a Dionex LC system to the modified Thermo Scientific Orbitrap Elite instrument. For each run, 5 µg of a 1:1 K-Ras•GppNHp:Raf_{RBD} solution (5 µL injection volume) in 50 mM ammonium acetate containing 5 μM magnesium acetate (pH 7.8) was injected onto a 2.1 mm × 150 mm ACQUITY UPLC Protein BEH SEC column with a 200 Å pore size and 1.7 µm particle size (Waters, Milford, MA). An isocratic mobile phase of 50 mM ammonium acetate at pH 6.8 was flowed at a rate of 100 µL min^{-1}. The LC effluent was introduced into the MS using a HESI source with an applied voltage of 4 kV. ESI-MS spectra were collected with a resolving power of 240 K at *m*/*z* 400. Normalized relative abundances of K-Ras•GppNHp + Raf_{RBD} complexes were calculated from peak areas of EIC traces of *m*/*z* values corresponding to each species.

Table S1. Table of theoretical and measured monoisotopic masses for each observed dimer in Figure 1. All expected masses account for one divalent Mg^{2+} ion bound per monomer subunit. The reported standard deviations represent triplicate data.

Mass (Da)		GDP + GDP	GppNHp + GppNHp
WT	Theoretical	*39511.327*	*39669.292*
	Measured at 20 µM	*39513 ± 1*	*39668 ± 1*
	Measured at 80 µM	*39511.7 ± 0.4*	*39668.9 ± 0.8*
G12C	Theoretical	*39603.287*	*39761.252*
	Measured at 20 µM	*39603.7 ± 0.4*	*39761.6 ± 0.5*
	Measured at 80 µM	*39603.1 ± 0.5*	*39760.8 ± 0.9*
G12V	Theoretical	*39595.407*	*39753.372*
	Measured at 20 µM	-	-
	Measured at 80 µM	*39593 ± 2*	*39753.1 ± 0.9*
G12S	Theoretical	*39571.347*	*39729.312*
	Measured at 20 µM	*39571.3 ± 0.2*	*39729.1 ± 0.5*
	Measured at 80 µM	*39571.3 ± 0.6*	*39729.9 ± 0.7*

Figure S1. Sequence of (A) WT K-Ras4b (1-169) and (B) the Ras binding domain (RBD) of Raf. Residues are numbered from the first glycine (G) of K-Ras4b in subsequent figures. The G12X position mutated to Cys, Val, or Ser is shaded in a gold box. (C) Structures of the guanosine phosphate ligands (1) guanosine 5'-diphosphate (GDP) and (2) guanosine 5'-[β,γ-imido]triphosphate (GppNHp).

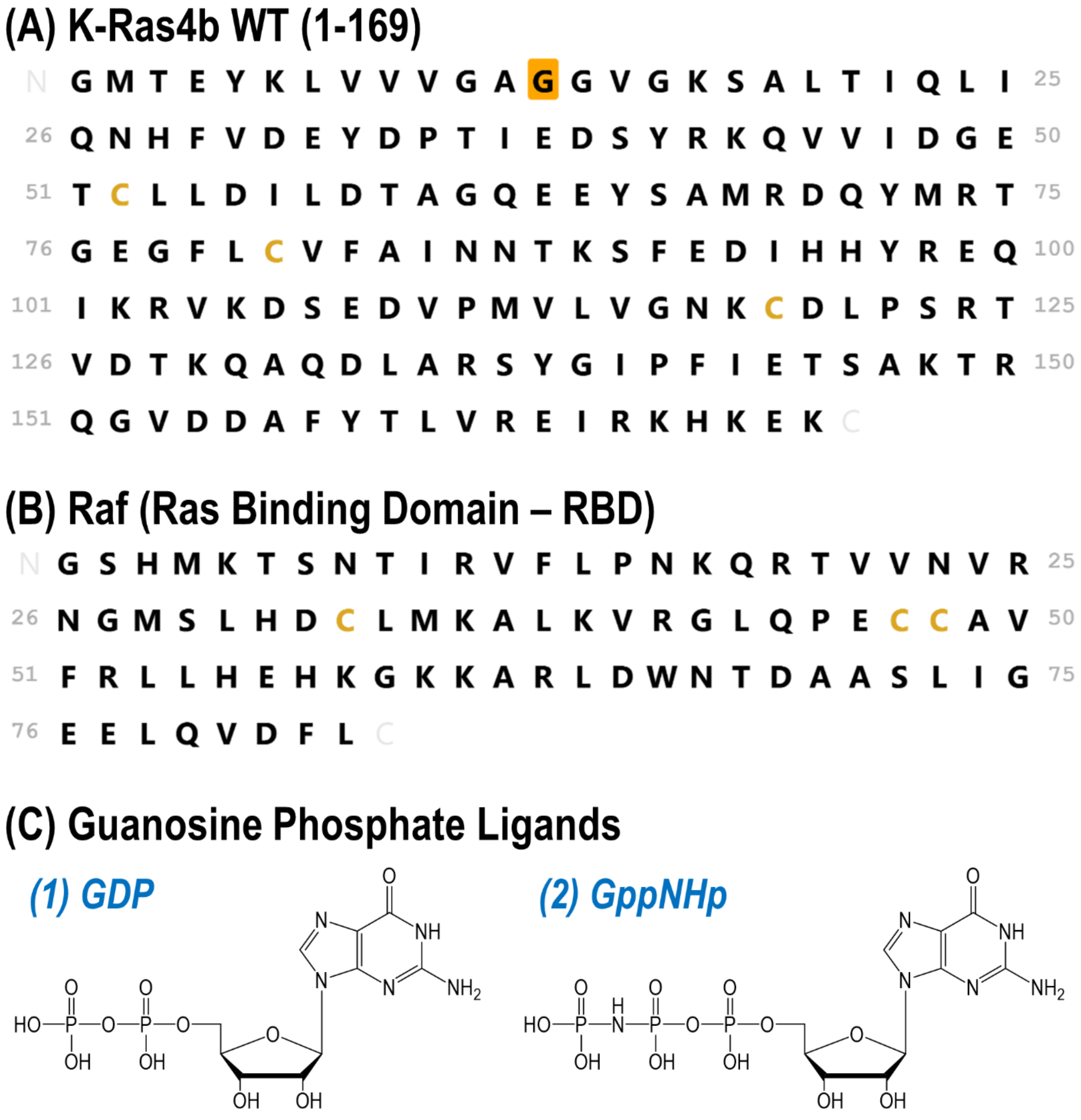

Figure S2. (A) Log of calculated *p*-values per residue for the differences plotted in **Figure S15**. The black dotted line represents a confidence threshold at 99%. (B) Histogram of all calculated *p*-values in (A-C) with the cumulative percentage shown in blue. Pooled standard deviations were used to perform Student's *t*-test for comparison of UVPD of each K-Ras variant to the WT. Assuming a two-tailed hypothesis, *p*-values were determined from calculated *t*-values. In summary, a *p*-value smaller than 0.01 at a given residue for a variant of K-Ras indicates that the average measured UVPD intensity within a triplicate measurement is statistically different from the measured average of the WT at the 99% confidence level.

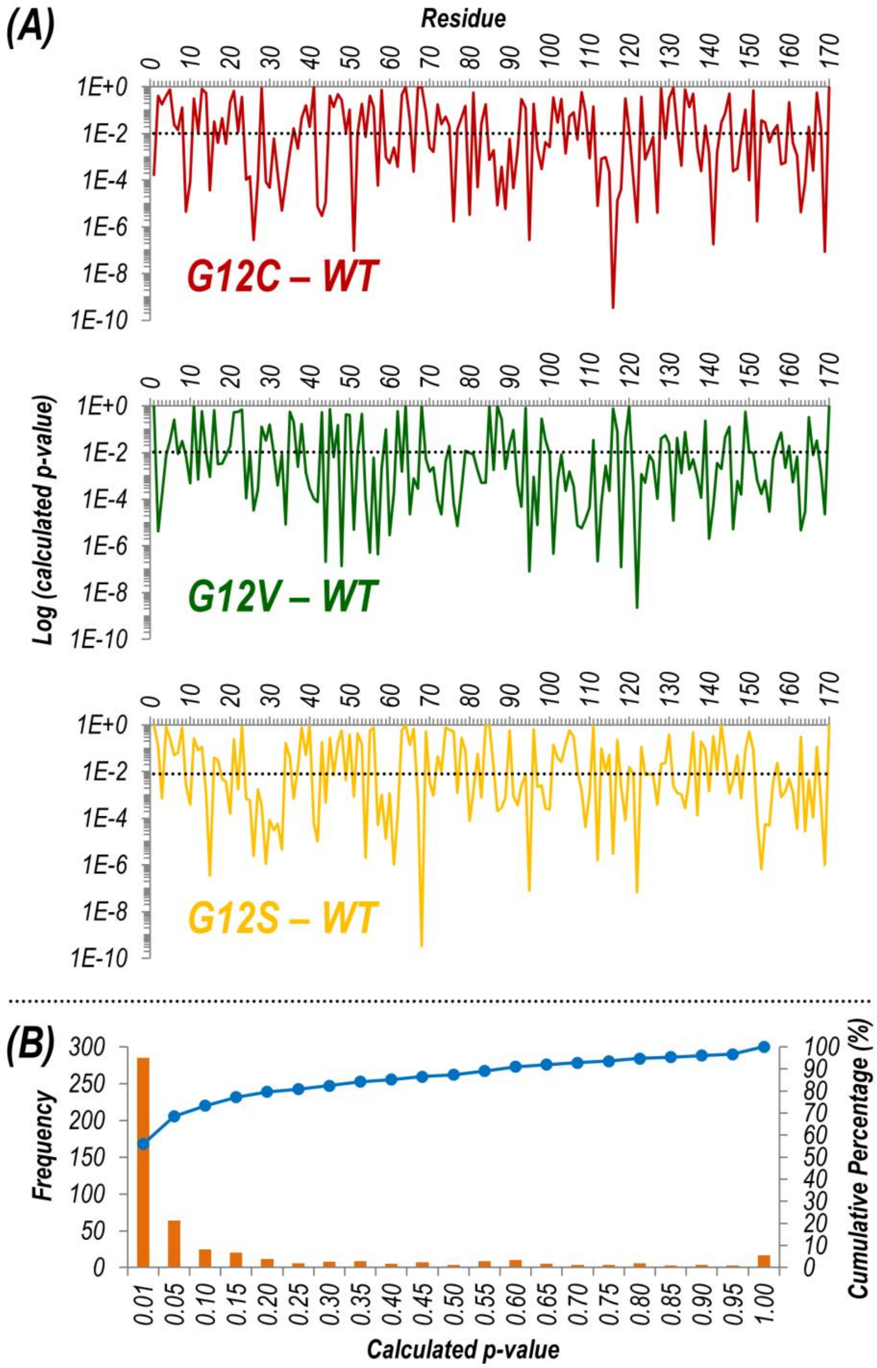

Figure S3. (A) Crystal structure of WT K-Ras bound to GppNHp and complexed with Raf_{RBD} (PDB ID: 4G0N) highlighting important loop and α-helical regions. The two possible dimer interfaces include the α-interface at α-C and α-D helices (shaded yellow) and β-interface at Switch I (shaded purple). Binding with most upstream or downstream effector proteins occurs at the stronger β-interface, but the α-interface might be used for homodimerization. (B) Secondary structure map of the catalytic domain of the Ras family of proteins. Isoforms are almost identical in these regions but differ significantly in the C-terminal hypervariable region (HVR). For this study, only residues 1-169 were examined.

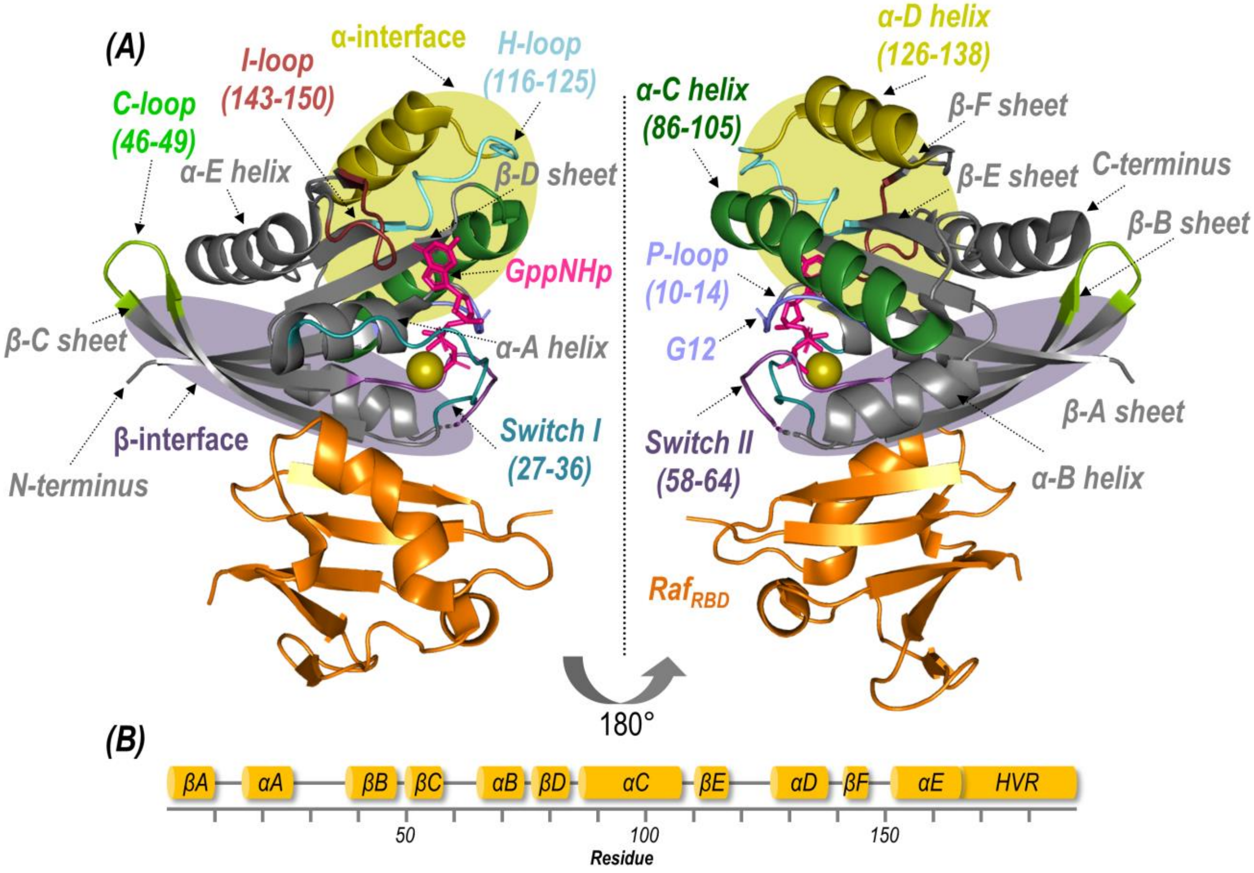

Figure S4. Native ESI mass spectra of (A) WT, (B) G12C, (C) G12V, and (D) G12S K-Ras bound to GDP (top) or GppNHp (bottom) (1:1 protein:ligand ratio) at a protein concentration of 20 μM or 80 μM. Observed species are labelled in panel (D, right-side) (9+, 10+, 11+ monomers and 12+, 13+, 14+ homodimers). A bar graph displaying the abundance of each observed homodimer normalized to the TIC is given in Figure 1. The theoretical and measured monoisotopic masses for each observed homodimer are reported in Table S1.

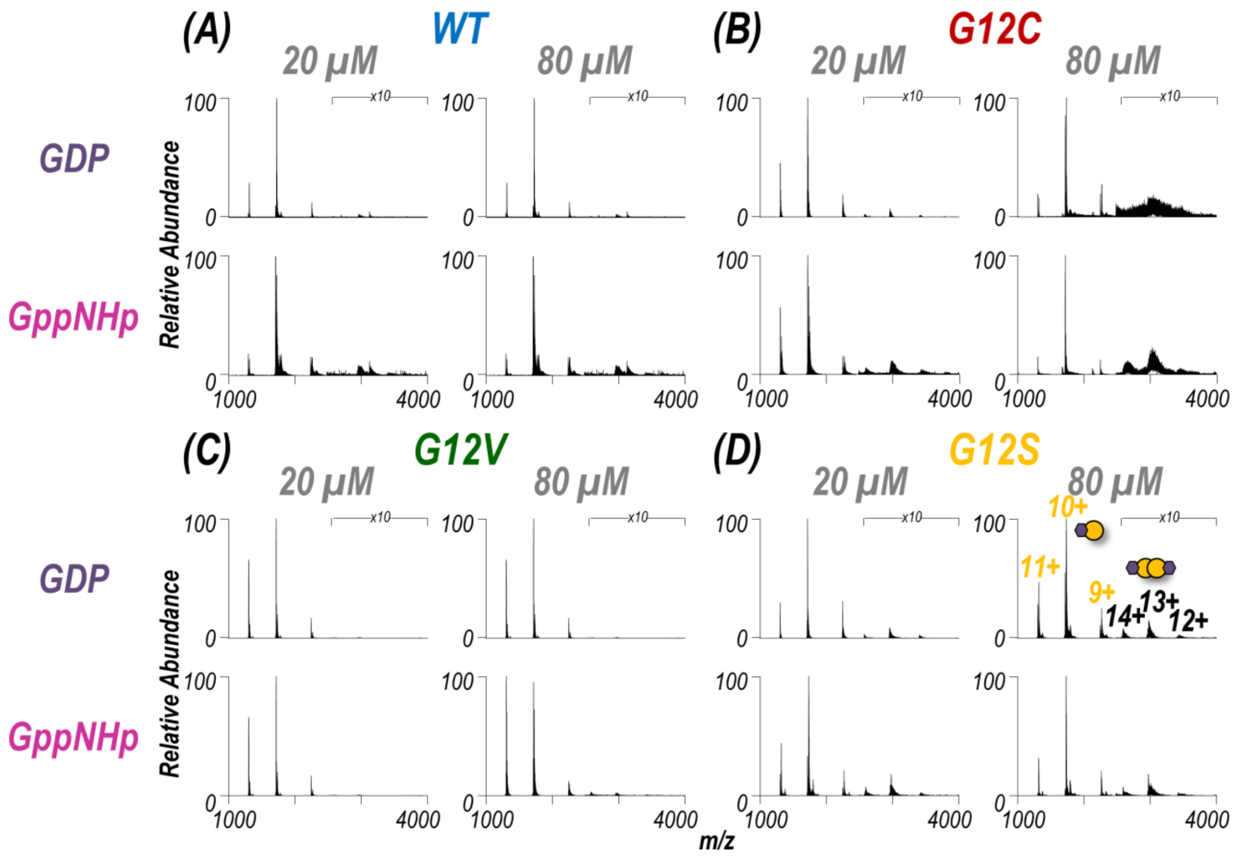

Figure S5. ESI mass spectra of the non-hydrolyzable mimics of GTP: (A) GppNHp and (B) GTP-γ-S sprayed in 50 mM ammonium acetate (pH 7.8). The spectrum for GTP-γ-S suggests the ligand is mostly hydrolyzed to GDP.

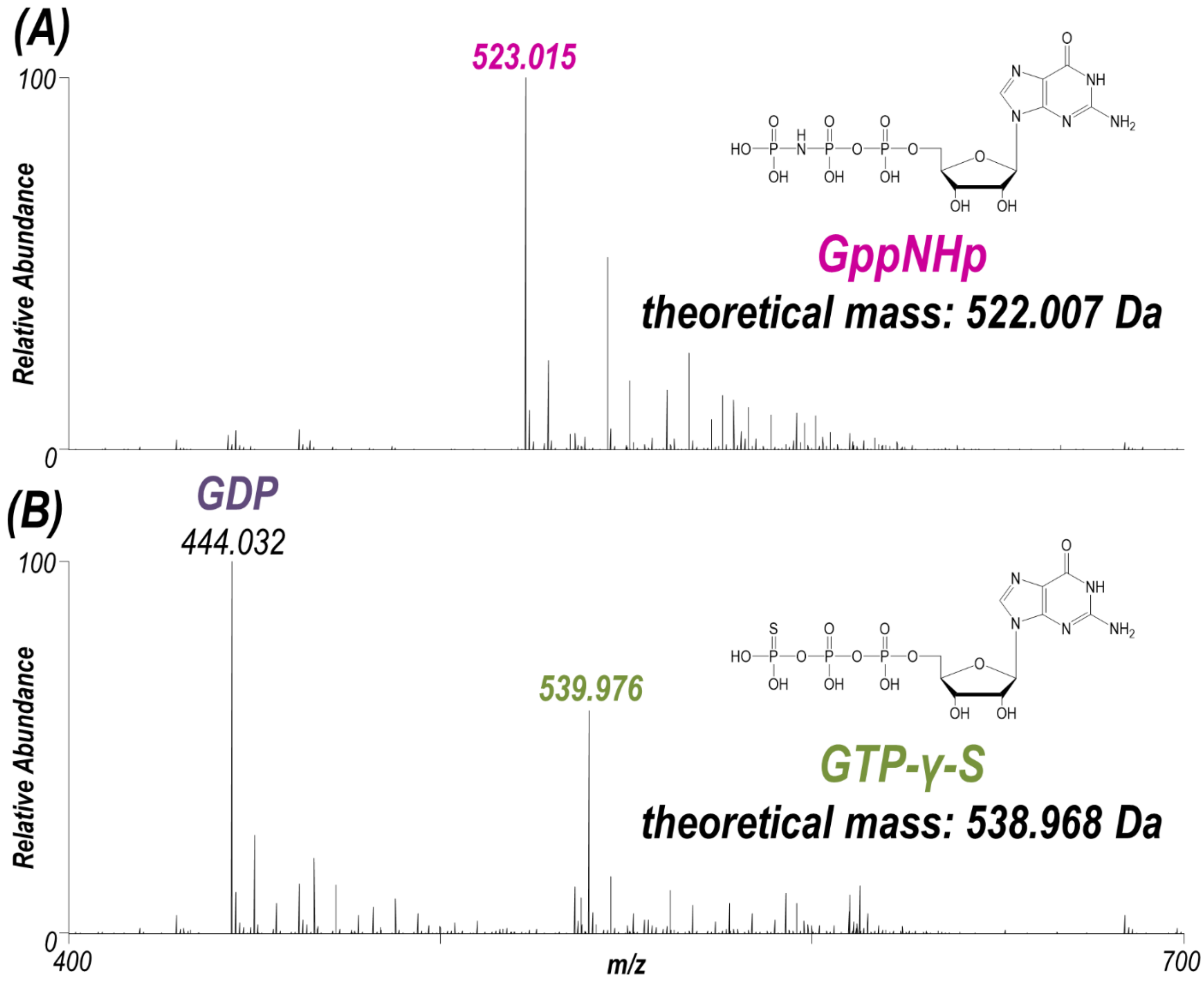

Figure S6. Native ESI mass spectra of (A) G12C, (B) G12V, and (C) G12S K-Ras bound to GDP (top) or GppNHp (bottom) (1:1 ratio) with an equimolar amount of WT K-Ras present in solution (total protein concentration of 20 µM or 80 µM). Observed species are labelled in panel (A, top right) (9+, 10+, 11+ monomers of each variant). No dimers were observed under these conditions. Insets show zoomed-in views of 10+ charge states of each variant of GDP-bound K-Ras (*m/z* 1970–2000).

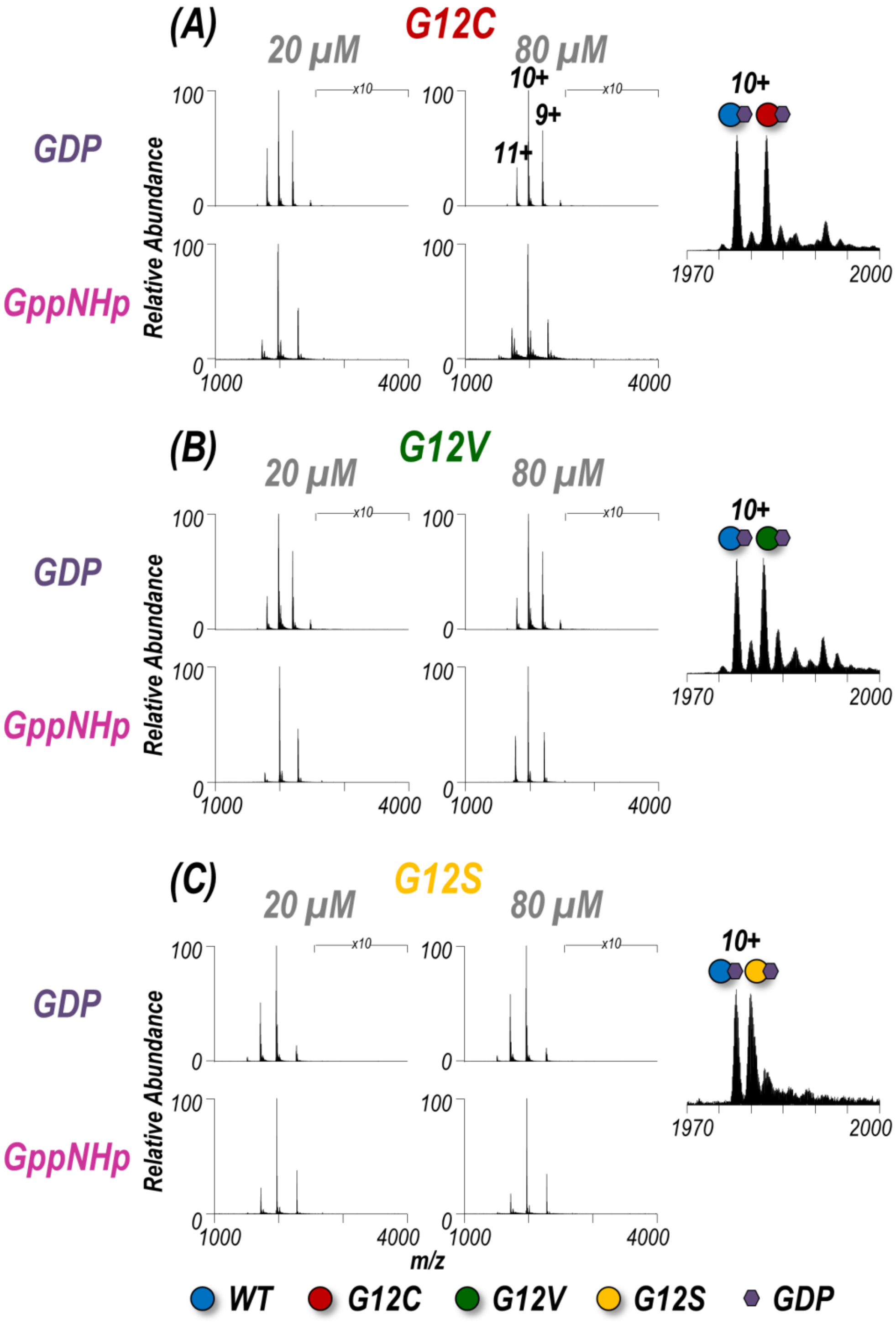

Figure S7. Native ESI mass spectra of 1:1 K-Ras:Raf$_{RBD}$ solution of (A) G12C, (B) G12V, and (C) G12S K-Ras bound to GDP (top) or GppNHp (bottom) (1:1 ratio). Observed species are labelled as colored spheres (6+, 7+, 8+, 9+ Raf$_{RBD}$ monomers, 9+, 10+, 11+ K-Ras monomers, and 10+, 11+, 12+, 13+, 14+ heterodimers). Corresponding spectra for WT K-Ras are given in Figure 2. K-Ras + Raf$_{RBD}$ heterodimers were only observed for solutions containing GppNHp, not GDP. Very low abundances of G12C and G12S homodimers are also observed. The relative abundances of each species are estimated from SEC-MS and reported in Figure 3. The 12+ heterodimer was selectively isolated and activated to yield the UVPD spectra shown in Figure S10.

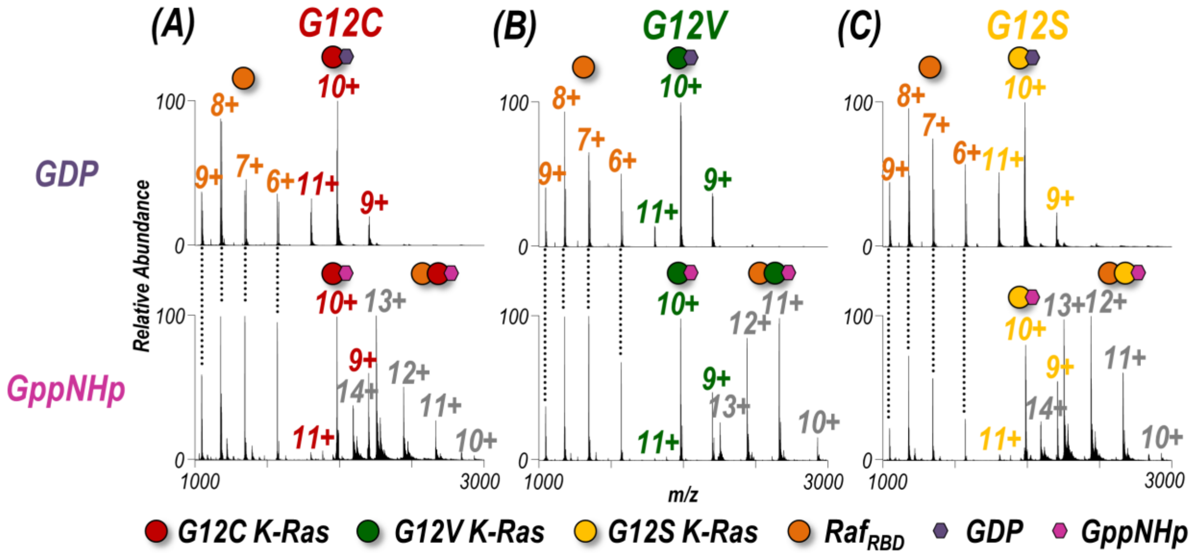

Figure S8. EIC traces (2.0–6.0 min) of the *m*/*z* values corresponding to K-Ras•GppNHp (red), Raf_{RBD} (blue), and K-Ras•GppNHp + Raf_{RBD} heterodimer (green) from on-line SEC of solutions containing WT, G12C, G12V, or G12S K-Ras•GppNHp with an equimolar amount of Raf_{RBD}. Peak areas were used to calculate the percent normalized relative abundance of each species reported in the bar graph in Figure 3.

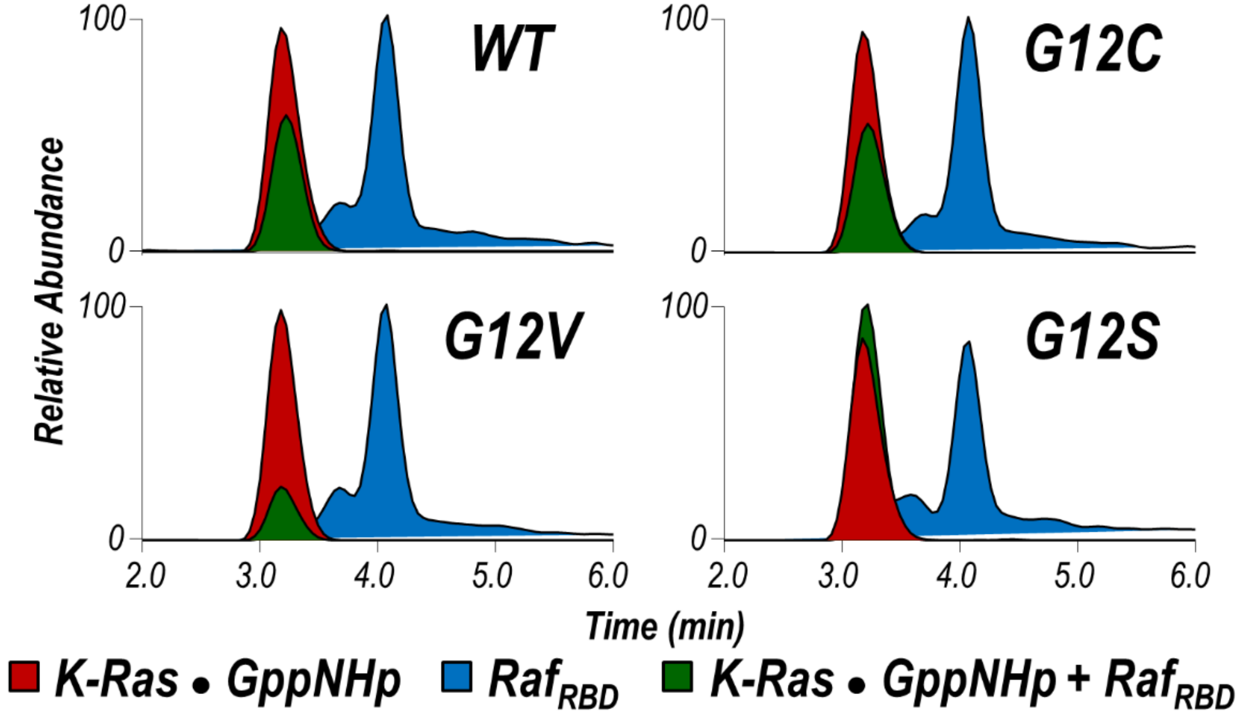

Figure S9. (A-D) Deconvoluted mass spectra of the K-Ras•GppNHp + Raf_{RBD} complexes (12+ charge state) selectively isolated for activation by UVPD. The isolated species activated are fairly homogenous with up to two Na adducts present for each variant.

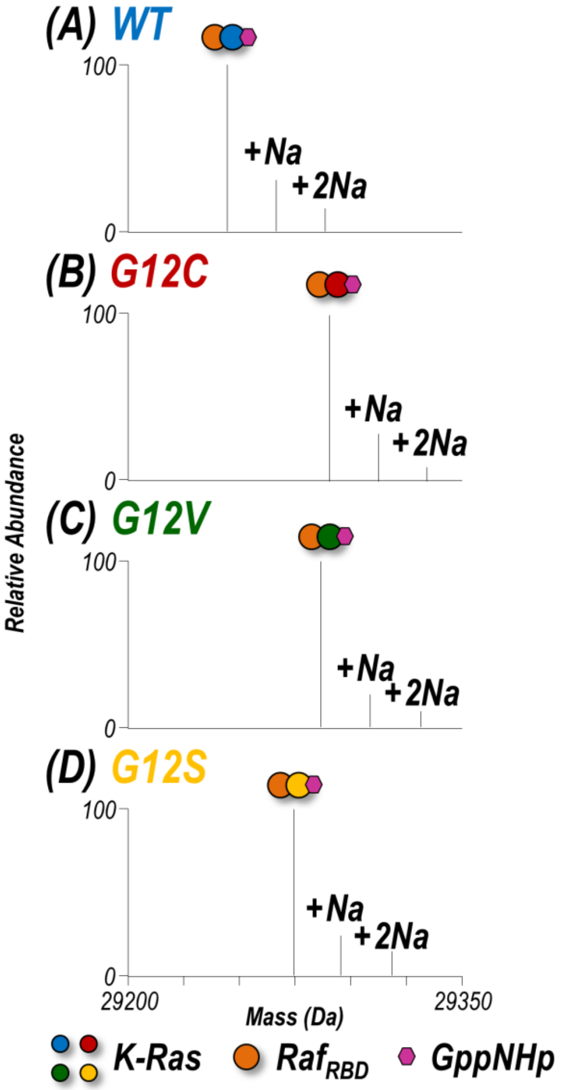

Figure S10. (A-D) UVPD mass spectra of the 12+ charge state of K-Ras•GppNHp + Raf_{RBD} heterodimers activated using a single 3 mJ pulse. Selected fragment ions are labelled in the expanded inset (*m/z* 1300-1350) in panel (A). Intact subunits are labelled for K-Ras•GppNHp in the 9+ charge state or Raf_{RBD} in the 3+ charge state.

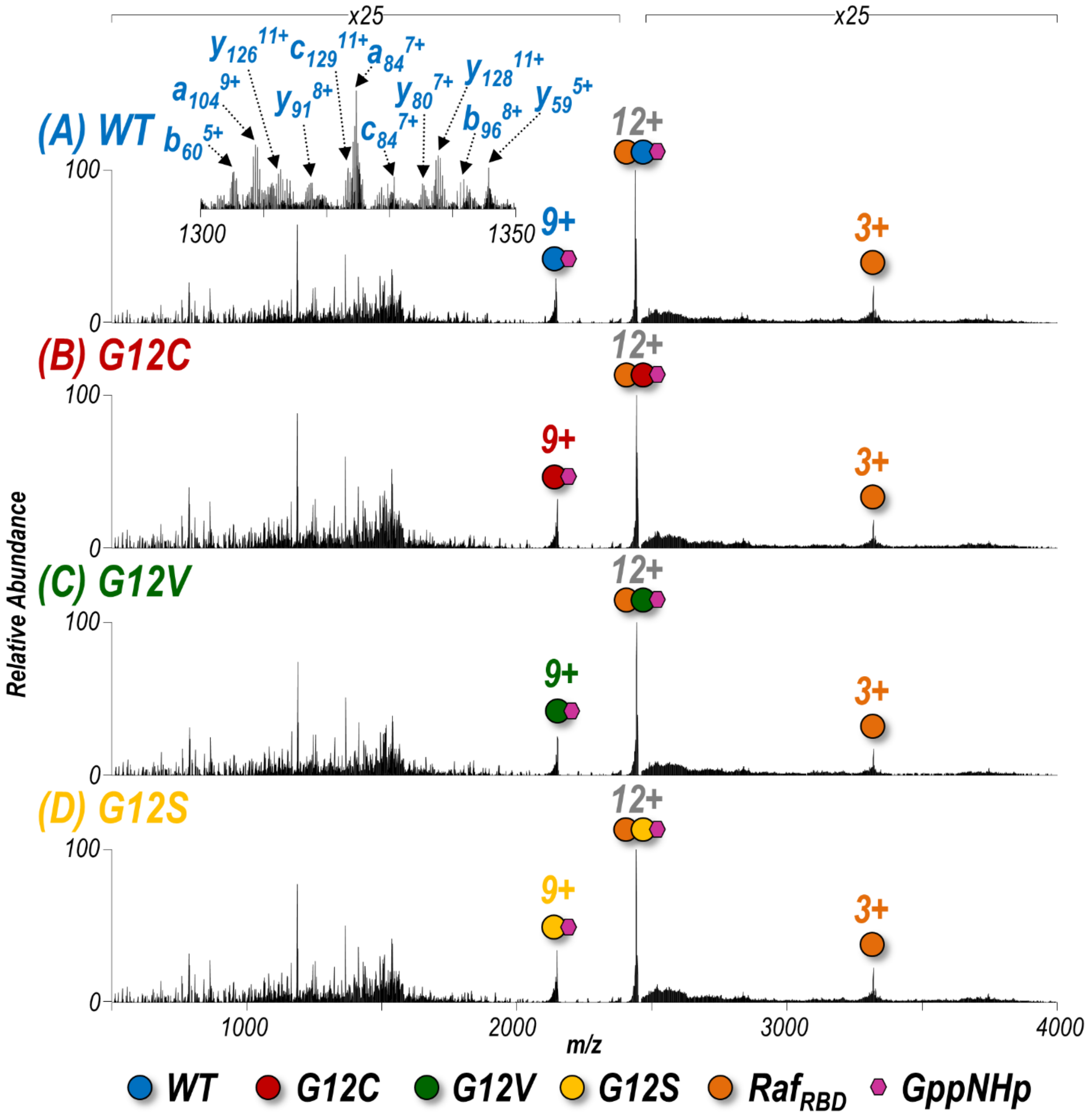

Figure S11. (A-D) Deconvoluted UVPD mass spectra (corresponding to the spectra in Figure S10) of all K-Ras•GppNHp + Raf$_{RBD}$ complexes (12+ charge state) using one 193 nm pulse at 3 mJ. Several abundant fragment ions are labelled in (A). Holo fragment ions (K-Ras•GppNHp bound to intact Raf$_{RBD}$) are denoted with ♦. The asterisks represent surviving precursor ions (non-deconvoluted) as an artifact during deconvolution.

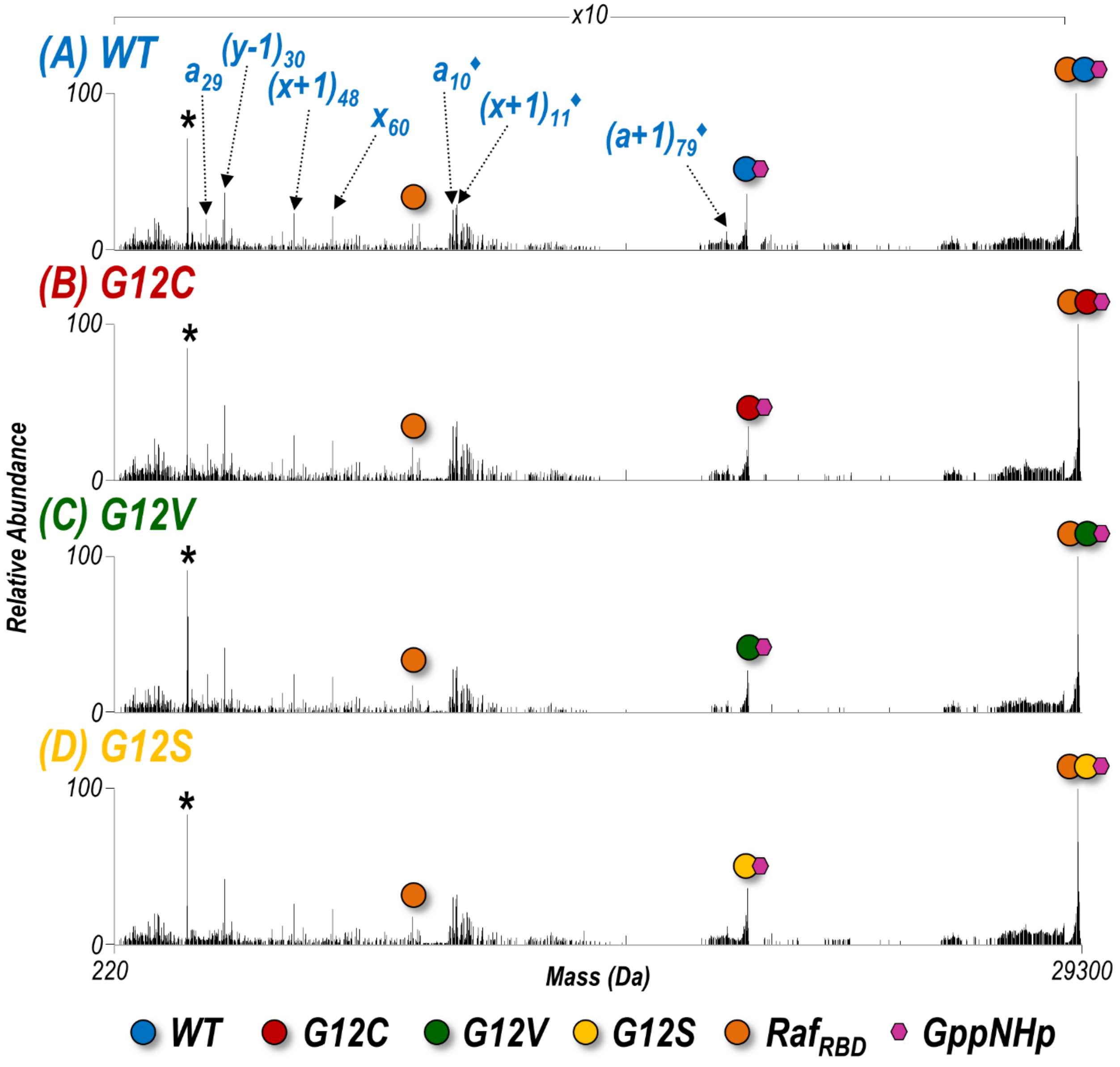

Figure S12. Sequence coverage maps of K-Ras based on identification of apo (ligand-free) fragment ions identified in the deconvoluted UVPD spectra shown in Figure S11 (12+ charge state of the K-Ras•GppNHp + Raf$_{RBD}$ heterodimer). The β-interface is shaded in purple. The apo ion sequence coverage was 85–89% for K-Ras in all complexes.

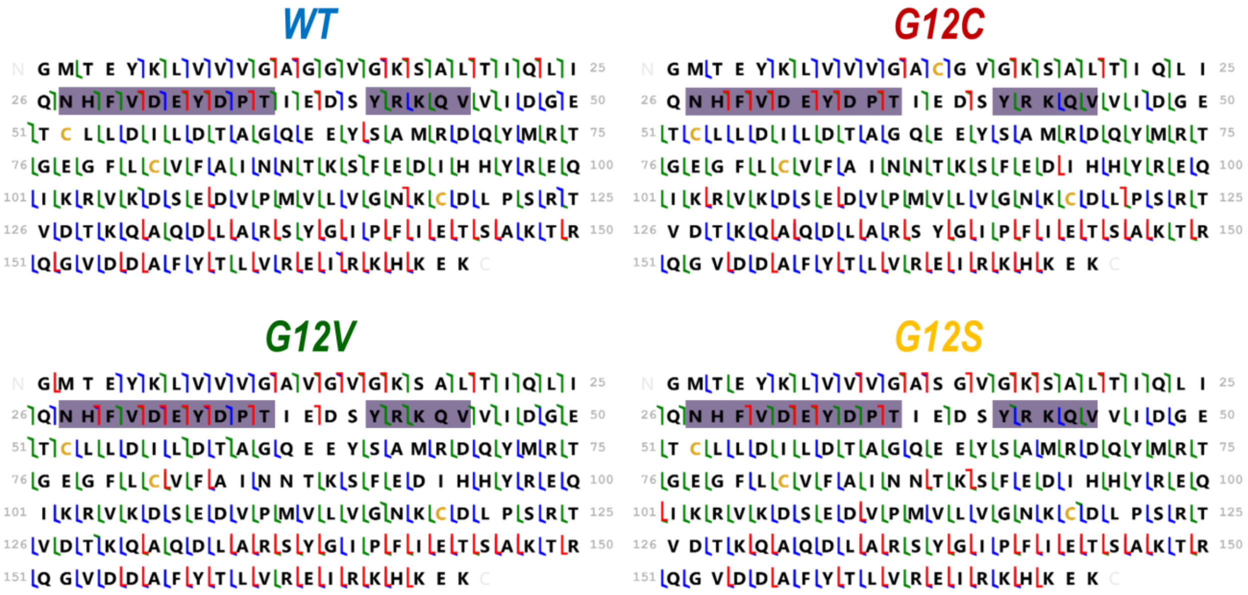

Figure S13. Crystal structures of K-Ras bound to GppNHp and complexed with Raf_{RBD} (PDB ID: 4G0N) with residues colored corresponding to backbone cleavages that produce N-terminal (*a*, *b*, *c*-type ions; blue), C-terminal (*x*, *y*, *z*-type ions; red), and bi-directional (green) holo fragment ions observed during UVPD of the 12+ heterodimer formed between (A) WT, (B) G12C, (C) G12V, and (D) G12S K-Ras and Raf_{RBD}. These structures correspond with the sequence shown in Figure 4A. The original UVPD spectra are shown in Figure S10. The GppNHp ligand (a non-hydrolyzable analogue of GTP) is shown as pink sticks and is labelled in (A).

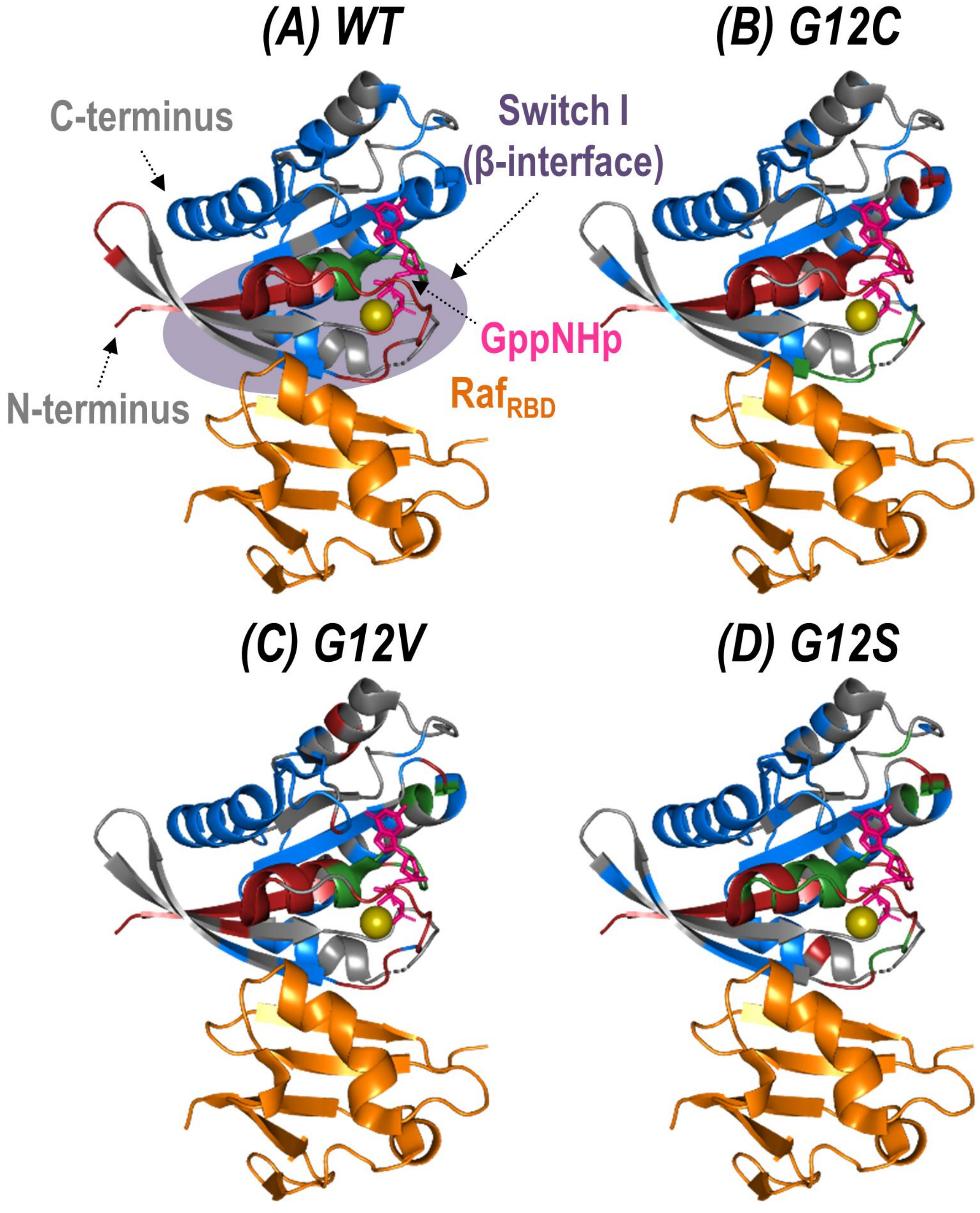

Figure S14. (A) Normalized TIC abundance of summed holo and apo product ions plotted per residue for each K-Ras•GppNHp + Raf_{RBD} heterodimer examined. These plots were used to create the difference plots shown in Figure S15 by subtraction of the values for each K-Ras variant from the corresponding values for WT K-Ras. (B) Heat map of the log of the values shown in (A). Relevant loops, α-helices, and interfaces are labelled underneath the *x*-axis.

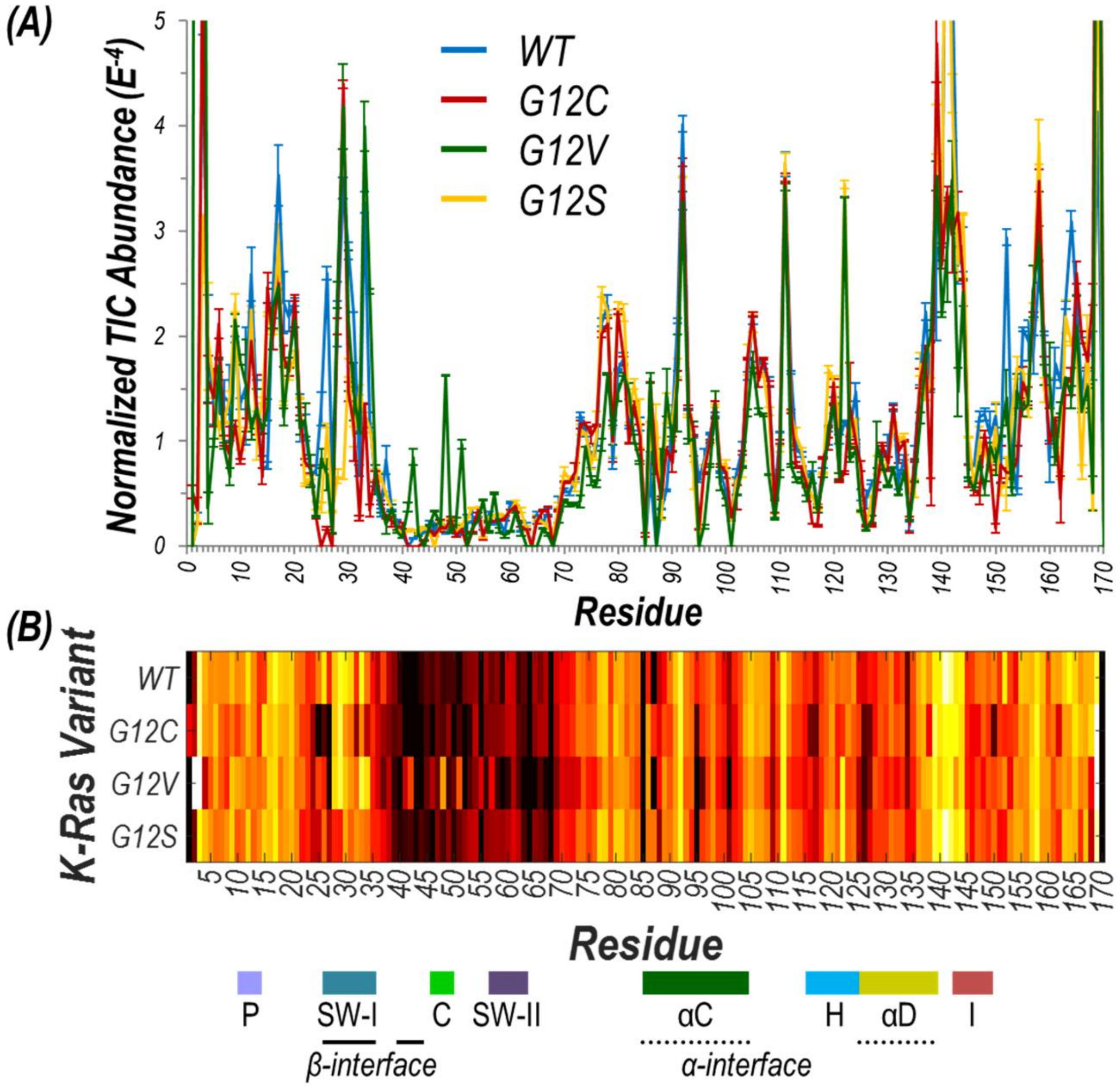

Figure S15. Difference plots showing the change in summed abundances of holo and apo fragment ions produced upon UVPD for Raf_{RBD}-bound heterodimers of (A) G12C, (B) G12V, and (C) G12S K-Ras variants compared to WT. The UVPD fragmentation plot for each of the complexes is shown in Figure S14. Relevant loops, α-helices, and interfaces are labelled underneath the *x*-axis, and the regions of particular interest (alpha interface, beta interface, and switch II) are bracketed for emphasis.

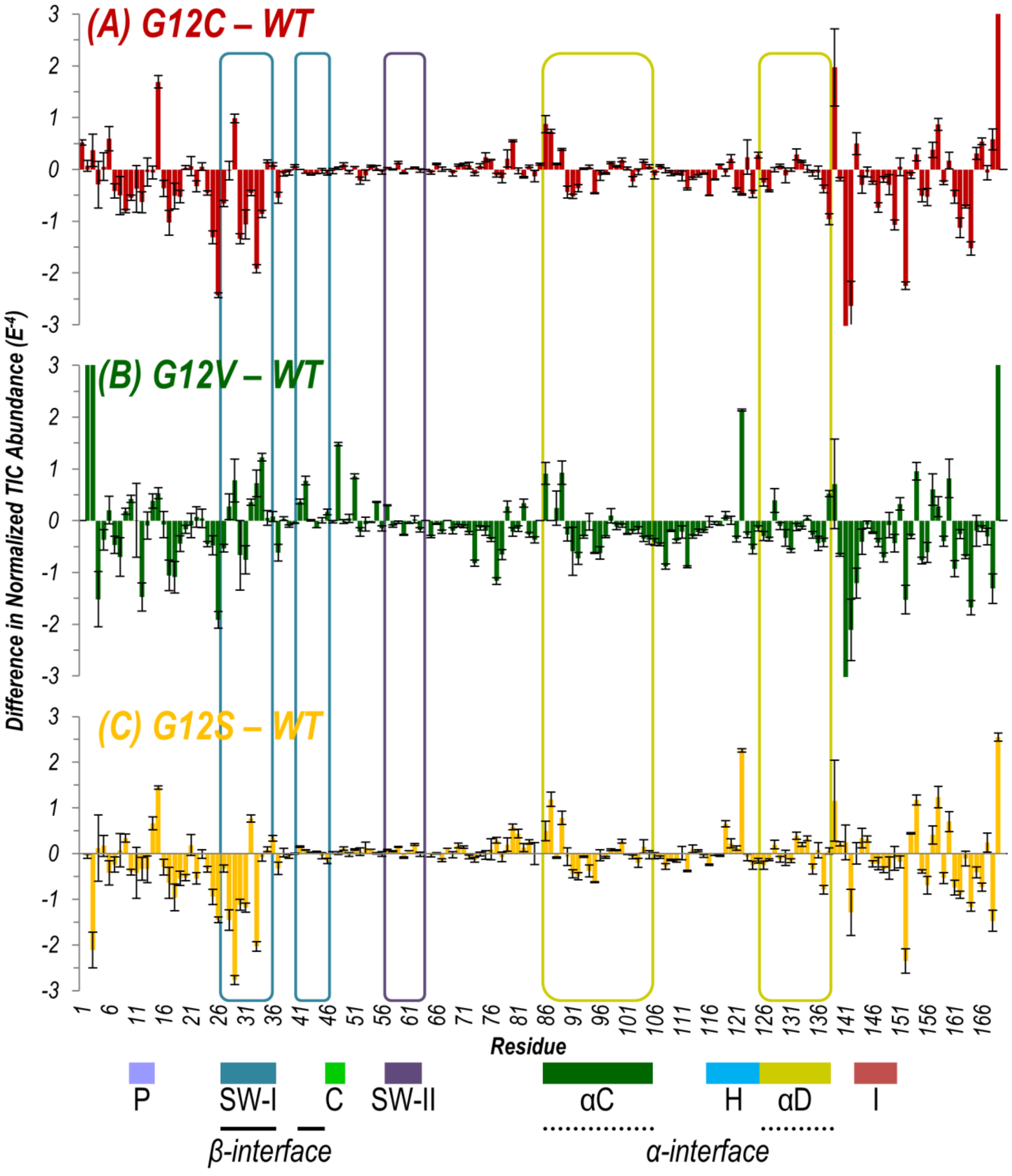

Figure S16. Expansions of the difference plots shown in **Figure S15** for Raf$_{RBD}$-bound heterodimers of G12C, G12V, and G12S K-Ras variants compared to WT for three key regions: β-interface (switch I), switch II, and α-interface (α-C and α-D helices). Asterisks indicate residues at which the summed abundance for the given variant is significantly different than the summed abundance for WT K-Ras at the 99% confidence level ($p \leq 0.01$). **Figure S2** gives plots of calculated p-values for all backbone positions. For the G12V variant, the difference plot is also shown considering only a/x-type fragment ions to demonstrate the same trends are observed when summing all ion types.

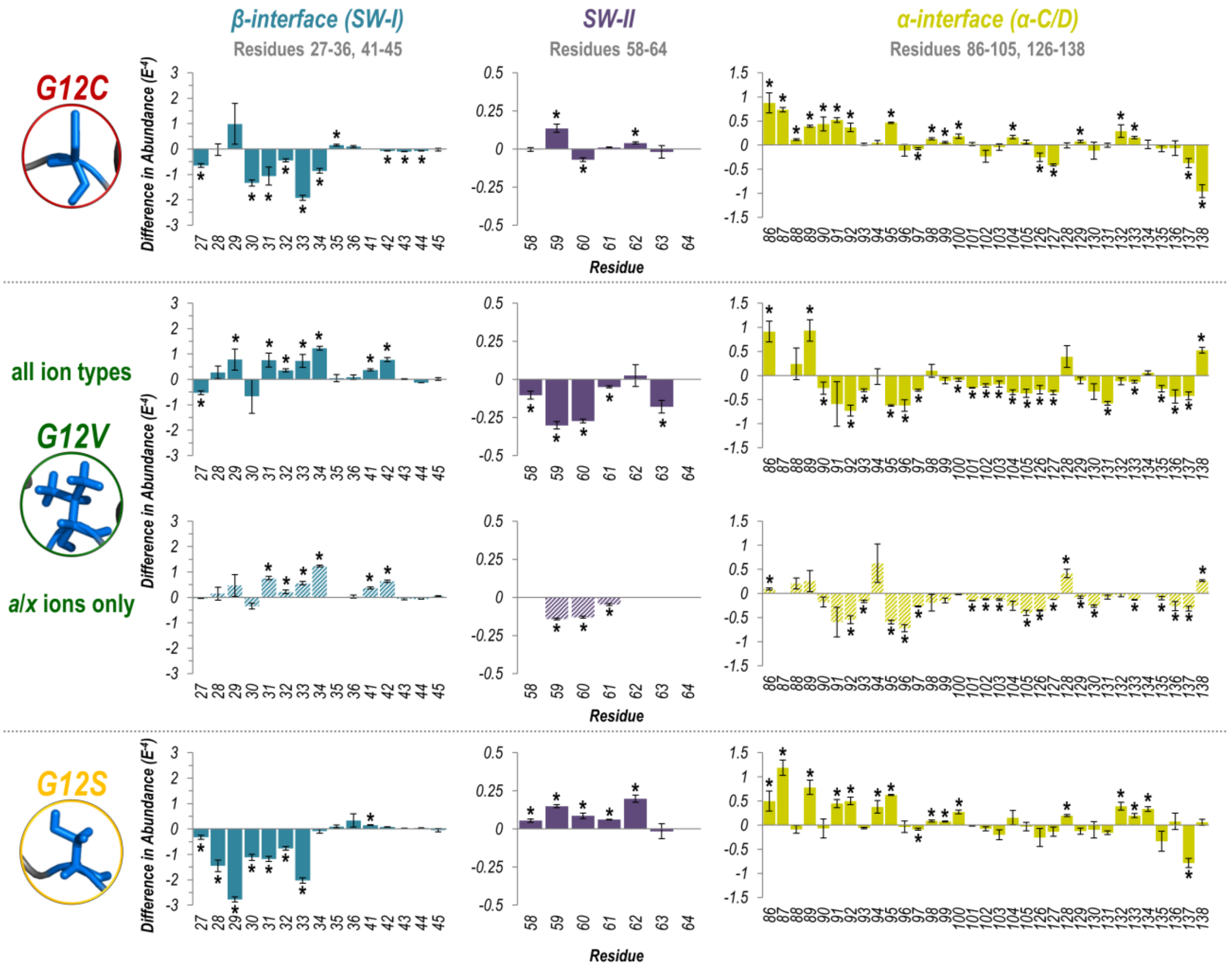

Figure S17. Summary of the observed enhancement (red) or suppression (blue) in UVPD backbone cleavage efficiency compared to the WT K-Ras for Raf_{RBD}-bound heterodimers of G12C, G12V, and G12S K-Ras in three key regions: β-interface (switch I), switch II, and α-interface (α-C and α-D helices). The difference plots from which these trends were determined are given in Figure S16.

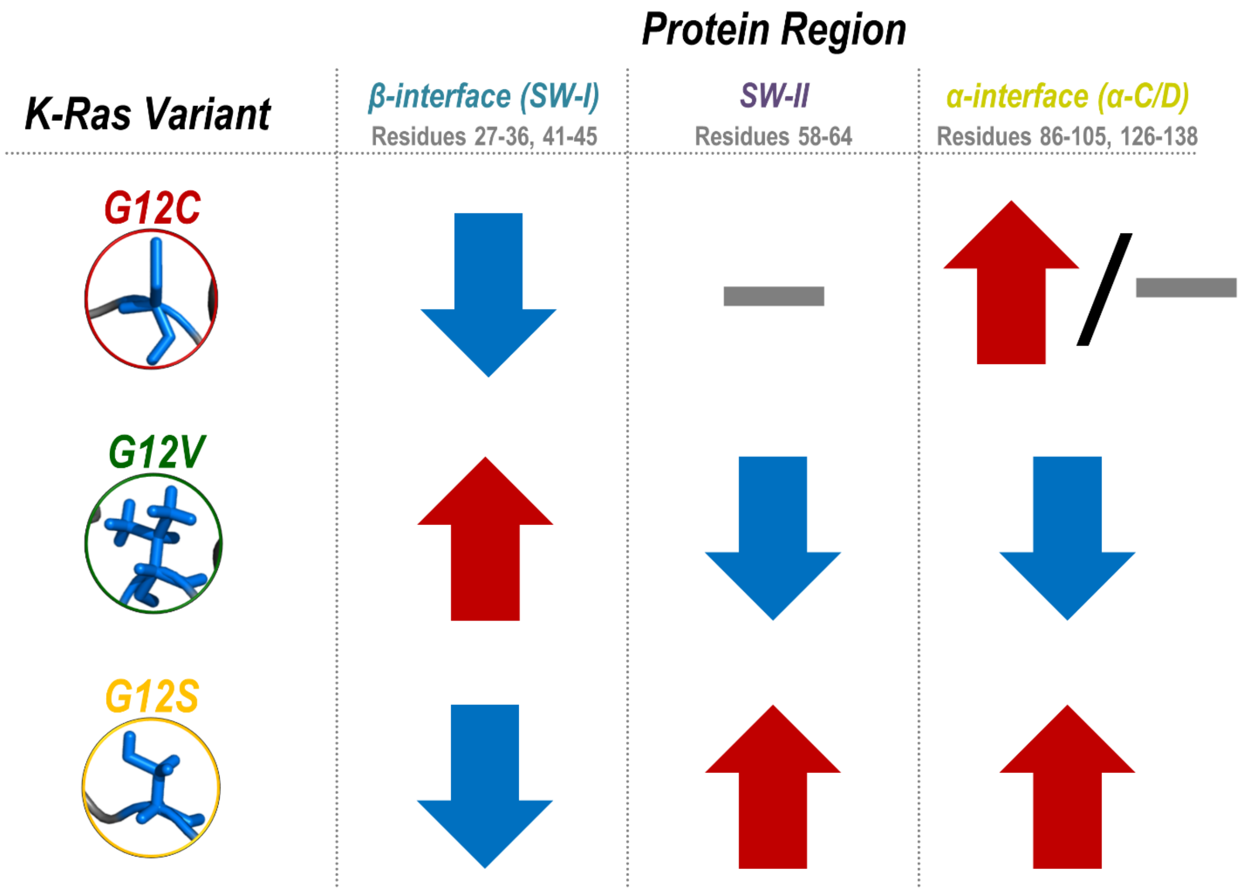

Supplement: Supplementary file 1 [file SC-010-C9SC01032G-s001.pdf]
